# Quantitative and spatially resolved detection of multiplexed microRNA from plant tissue via hybridization to hydrogel-bound DNA probes in nanoliter well arrays

**DOI:** 10.1038/s41378-024-00785-3

**Published:** 2024-10-08

**Authors:** Jennifer Fang, Patrick S. Doyle

**Affiliations:** Department of Chemical Engineering, Massachusetts Institute of Technology, Cambridge, MA USA

**Keywords:** Nanofluidics, Microfluidics

## Abstract

Understanding complex regulatory networks in plant systems requires elucidating the roles of various gene regulators under a spatial landscape. MicroRNA are key regulators that impart high information value through their tissue specificity and stability when using expression patterns for evaluating network outcomes. However, current techniques that utilize spatial multiplexing and quantitation of microRNA are limited to primarily mammalian systems. Here, we present a method to spatially resolve and quantify multiple endogenous microRNA in situ using ethanol fixed, paraffin embedded model plant species. This method utilizes target-specific microRNA capture along with universal ligating and labelling, all within functionalized hydrogel posts containing DNA probes in nanoliter well arrays. We demonstrate the platform’s multiplexing capabilities through analyzing three endogenous microRNA in *Arabidopsis thaliana* rosettes which provide useful answers to fundamental plant growth and development from the unique expression patterns. The spatial tissue technique is also validated using non-spatial small RNA assays to demonstrate the versatility of the well array platform. Our new platform expands the toolkit of spatial omics technologies for plants.

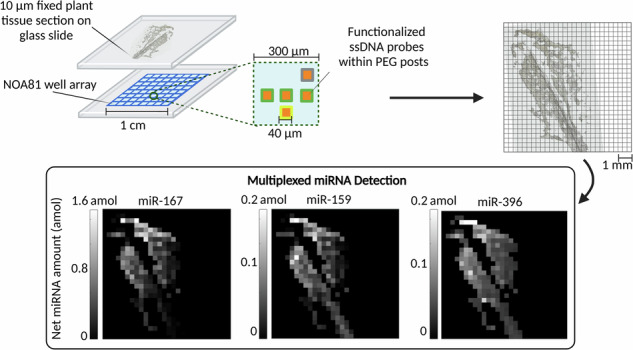

## Introduction

Studying the response and interactions of cell populations directly in the local cellular context is crucial to understand the tissue roles in model plant species^1,2^. The inter-cellular communication that directs the growth and development of plant tissue is driven by various gene regulatory networks which exist across the plant^2,3^. These complex gene regulatory networks are spatially regulated due to the tight cellular communication across the plant which creates distinctions in tissue morphology, making spatially resolving and multiplexing biomolecules present in regulatory networks a necessity^1,4,5^.

MicroRNA (miRNA) are short noncoding RNAs that function as post-transcriptional gene regulators. miRNA in plants have been shown to be dysregulated under various stressors and diseases, making them useful prognostic biomarkers for plant growth and response^6,7^. miRNAs are also stable in plant tissue in part because of the 2’-O-methyl moiety that is substituted to the 3’ end in vivo, protecting the miRNA from degradation^8,9^. The accumulation of stable miRNA in different cell types across the plant results in distinct spatial gene expression patterns due to the coordination between cells and high miRNA mobility to form unique expression gradients within tissues^2,10^. Moreover, when examining multiple plant miRNA using in situ hybridization, the accumulation patterns are reflected in high coordination both spatially and temporally^10,11^.

However, examining spatial miRNA patterns is limited in plants due to the inherent challenges when extracting miRNA from plant cells. These challenges within the cell include miRNA 2’-O-methylation which is ubiquitous in plant miRNA including *Arabidopsis thaliana* and has been shown to reduce ligase efficacy^8,12–14^. Furthermore, secondary metabolites which are abundantly found in plants can competitively bind to nucleic acids^15,16^. Plant cells also provide a barrier with carbohydrate-rich cell walls potentially hindering cell permeabilization and reducing the available miRNA transcripts^15,17^. Since plant characteristics often vary from tissue to tissue and require different assay changes to lyse cells and capture miRNA, tissue-specific features should be considered for optimizing extraction methodologies.

Due to the inherent challenges in spatially resolving biomolecules direct from plant tissue, limited approaches have been developed for the capture of miRNA. Techniques that capture single cell resolution while losing spatial information rely on enzymatically removing the plant cell wall for protoplast isolation^18^. Protoplasts are used as an input for fluorescence activated cell sorting (FACS) followed by conventional high throughput platforms (RT-PCR and microarrays)^19–21^. Despite the single cell information on the miRNA quantity and high degree of multiplexing, protoplast isolation techniques disrupt cells outside of their cellular context with ~90% retention^22^. Furthermore, miRNA-based microarrays have limited detection specificity because of base pair mismatches that arise from detecting similar miRNA sequences^23^. For RT-PCR, amplification biases can introduce errors due to the rigid design of templated miRNA capture probes and increased contaminate amplification during handling^24^.

There are also additional technical challenges involving the retention of spatial information in plant tissues. in situ hybridization methods such as fluorescence in situ hybridization and whole mount in situ hybridization using locked nucleic acid probes can be used to semi-quantitatively analyze the miRNA amounts with high specificity^2,11,25^. Limitations using in situ hybridization include restrictions on the number of unique transcripts (typically ~2) that can be multiplexed along with long experimental protocols^24^. Contemporary techniques to spatially resolve nucleic acids in plants explore high-resolution spatial transcriptomics through techniques such as barcoded microarrays (Visium, 10x Genomics)^15^ and sequencing (stereo-seq)^4,26^. These probe-based techniques contain a unique spatial barcode, unique molecular identifier (UMI), and poly(dT) region to generically capture target transcripts^27,28^. However, these methods involve hybridization of mRNA using the poly(dT) region after tissue permeabilization, making the techniques inherently incompatible to capture miRNA. Recently in situ polyadenylation of mammalian RNA has expanded the Visium platform to detect miRNA in mammalian tissues, however its application has been limited and has not been translated to plant tissue samples^29^.

This paper presents an optimized method for spatially resolved and quantitative, multiplexed detection of miRNA in plant tissue sections. This method expands on a protocol developed by our group for spatially detecting miRNA from formalin fixed and paraffin embedded (FFPE) mammalian tissue^30^. The technique uses sequence-specific DNA probes with patterned hydrogels for localized target capture on a nanoliter well array. We assess the intrinsic features of both plant cells and their miRNA to apply the optimized method on *Arabidopsis thaliana* leaf tissue, a model plant system. We then examine spatially resolved miRNA expression patterns by comparing the trends of endogenous miRNA patterns and then the relative amounts using bulk data.

## Results and discussion

### Spatial miRNA assay workflow using plant tissue

Our group has developed a platform using polyethylene glycol (PEG) hydrogel posts for multiplexed quantification of miRNA directly from unprocessed cells and FFPE mammalian tissue^30,31^. Prior work on using DNA probe functionalized PEG hydrogels has shown advantages for miRNA detection due to high probe-target binding specificity, non-fouling PEG substrate, and rapid solution kinetics during RNA hybridization^32,33^.

**Fig. 1:**
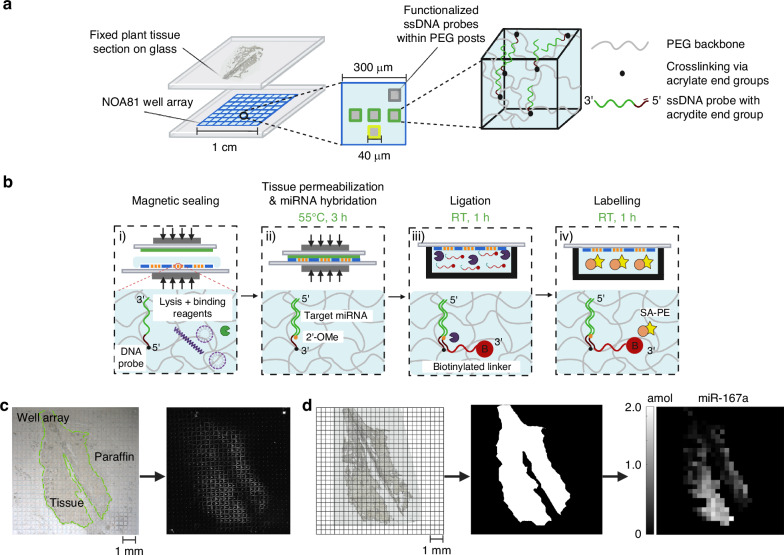
Well array and plant tissue assay workflow. Schematic of (**a**) plant tissue section over the nanoliter well array, followed by (**b**) miRNA tissue assay which involves (i) magnetic sealing of the tissue section and the well array along with hybridization buffer (sodium dodecyl sulfate, proteinase K, and PVP40) introduced to the wells (ii) lysis reagents in the hybridization buffer digest the tissue and lyse cells where free miRNA hybridize to the DNA probe within the hydrogel posts (iii) universal linkers facilitated by DNA ligase are ligated to the target miRNA and hybridize to the DNA probe. Excess linker is washed away and (iv) Streptavidin-R-Phycoerythrin (SA-PE) binds to the biotin, attaching a fluorophore at the binding site. After excess SA-PE is washed away (**c**) a fluorescent scanner images the captured miRNA to record the mean fluorescence in a post. **d** Representation of the tissue within wells is used to develop a digital mask to overlay on the fluorescent image

Hydrogel posts are sequentially fabricated using contact lithography within a nanoliter well array, allowing for spatial miRNA detection. PEG posts are copolyimerized with acrydite-modified DNA probes which contain a complimentary miRNA sequence for target specific capture (Fig. 1a). The relative spatial position of the post in the well translates to which DNA probe it is functionalized with. Following hydrogel post fabrication, a plant tissue section attached to a separate glass slide is mounted on the array with lysis and binding reagents within each well (Fig. 1a). We compress the fixed plant tissue and array together using magnets, such that the individual wells have isolated reactions (Fig. 1b). Reagents present during hybridization digest plant tissue, lyse cells, and bind to secondary metabolites for miRNA liberation. The freed miRNA are able to hybridize to the complimentary DNA probes within the hydrogel matrix. After hybridization, universal biotinylated linkers were ligated to the hybridized miRNA under a templated ligation reaction. Following ligation, excess linker was removed and streptavidin-R-phycoerythrin (SA-PE) bind to the biotinylated linkers to fluorescently tag the target-probe complex. SA-PE labelling results in a fluorescent footprint of the well array (Fig. 1c). The location of fluorescent posts are compared to the brightfield microscopy image showing the location where plant tissue is expected over the array (Fig. 1c).

Spatial information is retained by pixelating the average fluorescence from each post in every well into a 16-bit heatmap of pixel values (Fig. 1D). A digital mask of the tissue section is applied to the heatmap and by specifying a threshold (fraction of well occupied by tissue > 0.5) we can account for wells where partly filled tissue exist. Due to the flexibility of polymerizing posts with different capture probes we have developed a robust miRNA identification and quantification strategy allowing for spatial multiplexing (3+ endogenous miRNA). Adapting from our previous work, our assay addresses challenges when extracting miRNA in fixed plant tissue.

### Plant miRNA methylation

To optimize the performance of the plant tissue assay, we first sought to understand the differences between endogenous plant and animal miRNA. While plant and animal miRNA have the same biogenesis pathway, the methylation which occurs on the ribose of the last nucleotide (2’-OCH_3_) is universally present in plant miRNA in many species including *Arabidopsis thaliana*^9^. The function of miRNA methylation increases the stability by protecting the miRNA from subsequent degradation. A consequence of methylation is a decrease in ligase efficiency, which would decrease the number of active binding sites.

**Fig. 2:**
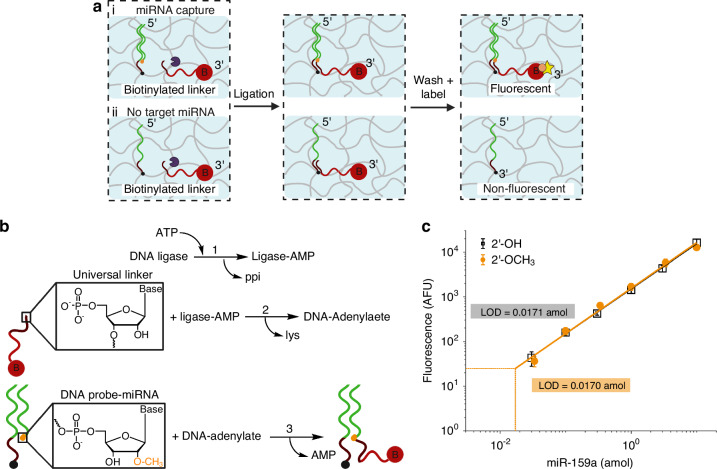
Ligase activity on methyl-substituted miRNA. Schematic of (**a**) ligation of the biotinylated linker when (i) miRNA is hybridized to the DNA probe and (ii) no target miRNA present. While miRNA is captured, the biotinylated linker ligates to the 3′ end of the target miRNA and washing retains the linker for labelling. When no miRNA is captured, linker attached to the DNA probe is washed away. **b** Mechanism for ligation using DNA ligase by (1) enzyme adenylation (2) transfer of AMP to the 5′ phosphate in the linker (3) DNA-adenylate covalently ligates to the DNA-miRNA probe. **c** Calibration curves with standard (grey) and 3′ methyl substituted (orange) synthetic miR-159a target and corresponding LOD. Solid vertical lines represent an LOD as three times standard deviation of control (0 amol) amount. Error bars represent one standard deviation

Prior evaluation of small-interfering RNAs (siRNAs) has shown faster hybridization rates with methylated nucleotides^34^. Any decrease in reported signal is expected to contribute by having lower enzyme activity during ligation. Due to the robust removal of excess linker and fluorophore (1 mL 1xTET) in our assay, only conjugated RNA-DNA would be fluorescently tagged (Fig. 2a). Figure 2b shows the general ligation mechanism using DNA ligase. Methylation in the probe-target complex does not actively participate in the DNA ligase-adenylate conjugation to miRNA, but the steric hindrances can limit attachment of the linker to the active site.

To understand methylation effects using the well array, we compared the limit of detection (LOD) between synthetic miRNA targets with a substituted 2′-O-methyl (2′-OCH_3_) modification to the last nucleotide. Since all plant miRNA are methylated at the 3′ end, we have the flexibility in selecting various miRNAs to investigate the effects of methylation. Therefore, we chose miR-159a as the target because the methylation has been well characterized across multiple species including *Arabidopsis thaliana*^35^. We also selected an internal control (0.2 μM biotinylated DNA probe for assay validity) and a negative control (cel-miR-54) in our panel (Table S1). cel-miR-54 was selected as the negative control since it is derived from *Caenorhabditis elegans* and is not naturally present in *Arabidopsis thaliana*. To determine a net fluorescence, the miR-159a signal was negative control subtracted and the reported fluorescence was then averaged using each endogenous post in the array. We discovered that our LOD for the methylated (plant) miRNA was 0.0170 attomole, which is nearly the same as the 0.0171 attomole LOD for the non-methylated (animal-mimic) miRNA (Fig. 2c). Hence, plant miRNA methylation does not adversely affect our assay LOD. Furthermore, our measured LOD for the 2′-OCH_3_ modified miRNA is the same order of magnitude found in our previous work (0.0081–0.053 amol) when studying several other miRNAs in animal systems^30^.

To understand why methylation of plant DNA does not change the LOD of our assay we consider related studies. First, a DNA-templated ligation scheme has been shown to be highly effective at using DNA ligase to covalently join 5′ DNA end to a 3′ end of a RNA strand (32 ligation events per minute) using a DNA template^36,37^. Furthermore, using single-stranded ligation, increasing reaction time with simultaneous reduction in reaction temperature along with increased enzyme concentration has completely recovered methylated small RNA targets^8,12^. Prior work in our group has also examined the effects of modifying ligation time and demonstrated that the majority of labelling events occur within 30 minutes of ligation (>98%) using excess DNA ligase^32^. Based on this prior research, it is reasonable to expect that our templated ligation assay is not adversely affected by having small RNA targets with 2′-OCH_3_ modification at the 3′ end.

### Assay optimization for miRNA detection in model plants

Prior to optimizing the assay for plant miRNA capture from tissue sections using the well array, we wanted to test for the natural variation of miRNA between adjacent tissue sections all examined with the same assay protocol. We used 10 µm thick leaf sections from 4-week-old Col-0 *Arabidopsis thaliana*. Sections adjacent from the same leaf were taken and the standard miRNA hybridization buffer (800 µg/mL Proteinase K and 2 w/v% sodium dodecyl sulfate) was introduced to the wells to measure miRNA amounts from the fixed plant tissue. We selected miR-167a as the target due to the high relative abundance in plant leaves, enabling greater flexibility for observing changes in the miRNA amount when examining modifications within the assay. Additionally, we used an internal control (0.2 µM biotinylated DNA probe for assay validity) and cel-miR-54 (endogenous to *Caenorhabditis elegans*) as the negative control (Table S1). Due to the natural abundance of biotin found in plant cells which can non-specifically bind to posts, the negative control is subtracted from the miR-167a signal during image analysis^38^.

**Fig. 3:**
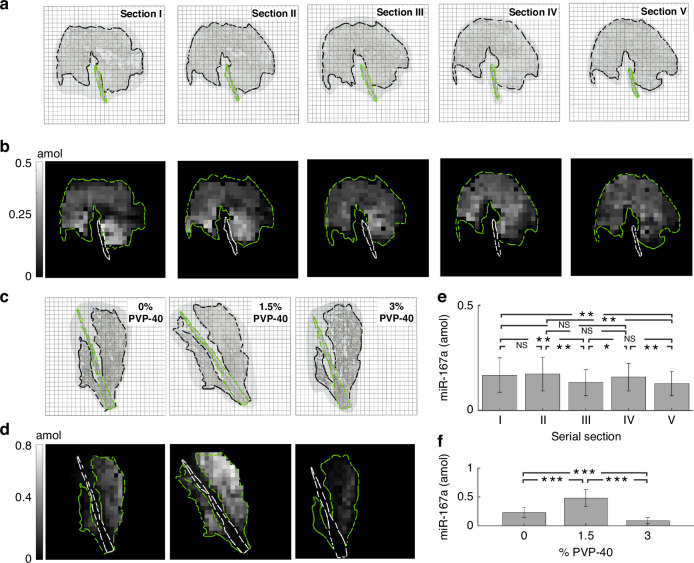
Optimization of tissue assay using serial sections and varying PVP40 concentration for miR-167a. **a** Location of each of the 5 adjacent tissue sections within the array where each box represents a 300 µm × 300 µm well, with well spacing separated by 50 µm. **b** Heatmaps for miR-167a of the 5 serial sections after performing the identical tissue assay. Each pixel in the heatmap corresponds to the amount of miRNA detected in a well. Reported values are negative control (cel-miR-54) subtracted. **c** Location of each of the 3 serial tissue sections within the array where each box represents a 300 µm × 300 µm well, with well spacing separated by 50 µm. 0%, 1.5%, and 3% PVP40 was added in the hybridization buffer alongside lysis reagents. **d** Heatmaps for miR-167a of the 3 serial sections after performing the tissue assay. Each pixel in the heatmap corresponds to the amount of miRNA detected in a well. Reported values are negative control (cel-miR-54) subtracted. **e**, **f** Quantitative plots for each of the tissue sections. Values are averaged from the heatmap after applying a mask to threshold pixel values with partly-filled tissue sections. Each value represents the mean, error bars represent one standard deviation. NS indicates not significant, *(*p* < 0.05), **(*p* < 0.01), ***(*p* < 0.001) indicates statistical significance using unpaired t-tests

Fig. 3a visualizes the locations of the five serial sections overlaid on the microarray wells (background grid) that were tested while using identical assay conditions without removing any paraffin. For each section, the heatmaps that showed localized miRNA amounts were reported. Each section displayed high binding specificity evident in both the section outline and in areas without tissue (Fig. 3b). Reported values shown in Fig. 3e using serial sections showed standard deviations within the average amount (CV = 40–45%). The variability in the miR-167a amounts show intrinsic fluctuations either in the assay or in the tissue sections that need to be considered when optimizing the assay and comparing proximal sections with different assay conditions. We further demonstrated that section to section variation of miR-167a decreased when paraffin was removed either before or after hybridization (Fig. S1). These results demonstrated that paraffin occludes wells and removes fixed posts after hybridization, thereby increasing variability in miR-167a amounts without paraffin removal (Fig. 3e). We thus removed paraffin before and after hybridization in subsequent optimization experiments due to high post retention (98%) along with consistent miR-167a signal in proximal sections.

To improve capture capabilities for plant miRNA, we further optimized the cell lysis conditions during hybridization. After cell lysis, secondary metabolites found in plant cells tend to co-precipitate with nucleic acids and can affect the yield of miRNA captured^16,39^. With the inclusion of polyvinylpyrrolidone, M.W. 40,000 (PVP40) insoluble complexes are formed with secondary metabolites and are precipitated out, thereby maintaining RNA in solution^15^. For optimization, we examined the effects of combining the lysis reagents with PVP40. We used adjacent sections from the same leaf and examined the endogenous miR-167a signal after the addition of PVP40 in the hybridization buffer (Fig. 3c). As shown in Fig. 3d, with the addition of 1.5 w/v% PVP40 in the hybridization buffer, miR-167a amounts have increased using the same lysis reagent concentrations (2 w/v% SDS and 800 µg/mL Proteinase K). At 3 w/v% PVP40, the signal decreased. This decrease is presumably because larger amounts of PVP40 can inhibit enzyme activity^39^. Since reported averages shown in Fig. 3f have statistical significance (*P* < 0.001) with the addition of 1.5 w/v% PVP40, we add 1.5 w/v% PVP40 to the optimized hybridization buffer. It is noteworthy that with the addition of both 1.5 w/v% and 3 w/v% PVP40 the SDS did not precipitate out of the hybridization buffer. This is expected as PVP40 can serve as a stabilizing agent for the lysis reagents^40^.

We also sought to explore the effects on miR-167a signal when varying Proteinase K concentration. Depending on the plant system, there are varying amounts of carrier protein (Argonaute) that anchors miRNA^41^. Therefore, we need to optimize the amount of Proteinase K to effectively denature the Argonaute proteins prior to hybridization. When varying the Proteinase K concentration, we found no significant differences in miR-167a signal from 200 µg/mL to 400 µg/mL with fixed 2 w/v% SDS and 1.5 w/v% PVP40 (Fig. S2). There are statistical differences in miR-167a amounts when increasing the Proteinase K concentration from 200 µg/mL to 800 µg/mL. Though the difference in miR-167a amounts were all within a standard deviation, we optimized at 800 µg/mL Proteinase K because qualitatively the increase in Proteinase K softened the tissue section preventing undigested plant matter to occlude the wells after hybridization. Proteinase K activity was also evaluated by modifying tissue digestion times fixed at 55 °C prior to Proteinase K deactivation (80 °C for 15 min) (Fig. S3). After increasing the digestion time from 15 min to an hour, there is a statistically significant (*P* < 0.01) increase in miR-167a amounts. Further increasing the tissue digestion time dries out the wells along the periphery of the array such that a 1-hour digestion time prior to 3-h miRNA hybridization is optimal.

### Multiplexed plant miRNA detection

After optimizing the assay for the model plant species *Arabidopsis Thaliana*, we examined the multiplexing performance using three endogenous plant miRNAs. The three plant miRNA in the panel (miR-167a, miR-159a, miR-396b) were selected because they are highly conserved between different plant species and express unique expression differences under growth and development. The role of these miRNA ultimately affects the leaf size and shape^42^.

To show high binding specificity during multiplexing, we determined the cross-talk for each target miRNA in the panel. In every well, we polymerized five posts where three posts target a unique miRNA (miR-167a, miR-159a, miR-396b) along with an internal and negative control (Table S1). Using a buffer with synthetic target added (no lysis or binding reagents), we spiked 0.3 amol of one miRNA target separately to the wells and sealed with a glass slide using magnets before hybridization. The resulting signal after performing the assay was control (0 amol) subtracted, and the matched signal relative to each target was calculated (Fig. S4). The average signal cross talk from Fig. S4 was 1.5% where the maximum signal cross talk was 3% when spiking in 0.3 amol of miR-167a. Prior work using targets with high sequence similarity (1-2 base pair mismatches) reported a maximum signal cross talk of 27% when comparing four members of the let-7 family^32^. The high specificity demonstrated in our panel shows low sequence similarity among the target plant miRNA.

Furthermore, calibration curves were constructed for each target miRNA by adding known synthetic amounts to each well and sealing the wells with a glass slide before hybridization (Fig. S5). The reported signal was control (0 amol) subtracted from the fluorescent amount from each post. The LOD for the endogenous miRNA in the panel was between 0.0036-0.0170 amol which is the same order of magnitude from previous work detecting miRNA from mammalian cells (0.0081–0.053 amol)^30^. Notably, in Fig. S5 when simultaneously adding 0.3 amol of three targets to one array (multiplexed), the averaged target signal is identical to the signal when adding 0.3 amol of one miRNA target to the wells (~500 AFU) (Fig. S4).

**Fig. 4:**
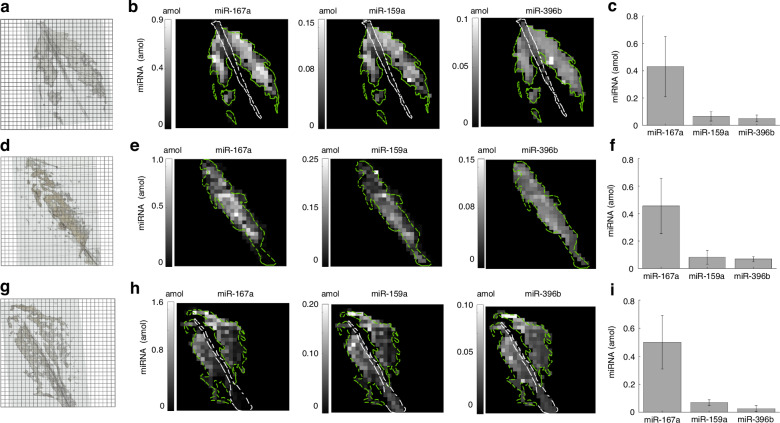
Multiplexed miRNA from plant tissue assay using different *Arabidopsis Thaliana* leaves. **a**, **d**, **g** Location of tissue section within the array where each box represents a 300 µm × 300 µm well, with well spacing separated by 50 µm. **b**, **e**, **h** Representative heatmaps for three plant miRNAs detected after performing assay after optimization. Each reported value is negative control (cel-miR-54) subtracted and relates to the miRNA captured in its corresponding 300 µm × 300 µm well. **c**, **f**, **i** Quantitative plots for each plant miRNA in the multiplexed assay from (**b**, **e**, **h)**. Values are averaged from the heatmap after applying a threshold to pixel values with partly-filled tissue sections. Each value represents the mean and error bars represent one standard deviation

After demonstrating high specificity with the endogenous miRNA in the panel, multiplex miRNA detection from ethanol fixed plant tissue sections was carried out. We used tissue from three different 4-week-old col-0 *Arabidopsis thaliana* leaves while targeting three different plant miRNAs (miR-167a, miR-159a, miR-396b) with one internal and negative control in the panel (Table S1). After fabricating the posts within the wells, the optimized hybridization buffer (1.5 w/v% PVP40, 2 w/v% SDS, 800 µg/mL ProK) was added and sealed with the fixed tissue section and magnets, and then imaged using brightfield microscopy (Fig. S6). Fig. 4a, d, g references the locations of the tissue sections within wells that were used during the assay. After performing the assay shown in Fig. 1b, a fluorescent footprint was produced (Fig. S6). The signal from the negative control (cel-miR-54) was subtracted from the unprocessed signal from each post and a heatmap for each endogenous miRNA was developed for each tissue section (Fig. 4b, e, h).

From the fluorescent footprint we observed that the biotin naturally found in the plant leaf non-specifically binds to the wells, conforming to the tissue location within the array (Fig. S6). The amounts of the endogenous miRNA show similar expression profiles between tissue sections from different plants (Fig. 4b, e, h). Furthermore, we averaged the miRNA amounts of wells containing tissue from Fig. 4b, e, h and reported the amounts of each endogenous miRNA as the net miRNA present in each tissue section (Fig. 4c, f, i). From Fig. 4c, f, i, we show a consistent net abundance from the same tissue section (amount: miR-167a > miR-159a > miR-396b). The higher relative expression levels in miR-167a compared miR-159a to in Col-0 *Arabidopsis thaliana* is consistent with prior studies using northern blot^43^.

Spatial observations of endogenous miRNA are highlighted when examining Col-0 *Arabidopsis thaliana* rosettes. From Fig. 4, we observe that miR-167a amounts are less expressed in the leaf vein compared to the blade. Using ISH, miR-167a was not detected in vascular tissues (veins) demonstrating that miR-167a is actively regulated in the vascular system which corroborates our findings. Furthermore, when evaluating spatial miR-396b profiles from Fig. 4, either expression homogeneity (Fig. 4b, e) or accumulation around the leaf perimeter (Fig. 4h) is observed. We validate our findings when using a GUS reporter gene assay as miR-396b reveals an expression gradient where the signal is highly conserved around the perimeter of a developing leaf. However, depending on the leaf age, signal homogeneity is observed for older leaves within the same plant^44,45^. We are able to demonstrate high signal specificity and miRNA conservation between different *Arabidopsis thaliana* plant leaves. Our adaptable technique can be expanded for different applications such as capturing miRNA from various plants to understand species-specific expression patterns or to study the different spatial patterns under a broad range of metabolic conditions. For instance, miR165/miR166 accumulates only in the periphery of the abaxial side of leaves due to the tight coordination across the growth axes, a phenomenon that is beginning to be understood^46^. Therefore, we can apply our method to adjacent sections across the entire leaf to clarify locations where miR165/miR166 is biologically relevant. From our detection platform, we can begin to learn and characterize the spatial miRNA expression profiles of numerous miRNAs, which can elucidate the developmental and regulatory roles of miRNA in precise tissue regions.

### Assay validation

**Fig. 5:**
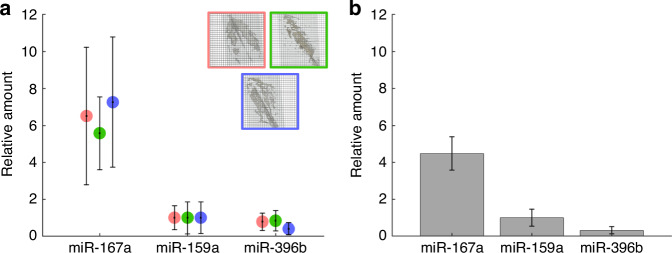
Assay validation through evaluating the well array assay performance. Relative amounts after multiplexing three endogenous miRNAs from *Arabidopsis thaliana* plant leaves using the (**a**) optimized tissue assay (**b**) bulk (non-spatial) assay measuring the extracted small RNA enriched fraction. The endogenous miRNA amounts in both the plant sections and small RNA enriched sample was normalized to miR-159a. The miRNA from the three plant leaf sections tested using the optimized tissue assay was individually normalized by miR-159a and reported. Error bars represent one standard deviation

To determine the optimized tissue assay performance, we compared the relative measured amounts to commercial methods for small RNA extraction and detection. Using the multiplexed, spatial endogenous miRNA amounts shown in Fig. 4, we determined the relative expression from each ethanol fixed, paraffin embedded section tested. The miRNA amounts recovered were normalized to the corresponding miR-159a levels since miR-159a has shown to have an intermediate abundance between miR-167a and miR-396b (Fig. 4), allowing for lower abundant transcripts (miR-396b) to be easily visualized. Furthermore, we reported the normalized miRNA values as a relative amount (Fig. 5a). For miR-167a the relative amount varies between 5.5-7 and for miR-396b the relative amount is between 0.39 and 0.78. Since sections were taken towards the center of the leaf with the greatest tissue surface area, both palisade mesophyll and spongy mesophyll cell types encompass the leaf center and are reflected in tissue section^47^. Therefore, from Fig. 5a we show the relative amounts taken from different leaves have consistent values around the same cross-sectional area.

We then compared the relative amounts in Fig. 5a to validate the tissue assay performance using commercial extraction methods with the well array. We used commerical cell lysing and spin column isolation techniques (RNAqueous micro kit) along with PVP40 (plant RNA isolation aid) to capture the small RNA enriched fraction from 4-week-old Col-0 *Arabidopsis thaliana* leaves. The collected small RNA fraction was validated using spectrophotometry (Nanodrop) and small RNA electrophoresis (2100 Bioanalyzer) tools to determine the quantity and purity of small RNA extracted (Fig. S7). Fig. S7 demonstrates that with the addition of 5 v/v% PVP40, the extracted RNA purity (A230/A260) increased from 1.1 to 1.2 (data not shown) to 1.84–1.9, indicating the successful removal of contaminates such as polyphenols present in plant tissue. After evaluating the quality of the small RNA extraction, we multiplexed three endogenous miRNA targets (miR-167a, miR-159a, miR-396b) with the addition of one internal and negative control. 0.032 ng of small RNA was added to the wells under ideal hybridization conditions for synthetic assays (350 mM 1xTET) before sealing with a clean glass slide and adding magnets^32^. The measured miRNA amounts were normalized to the miR-159a amount and reported as a relative amount (Fig. 5b).

From Fig. 5, the trends for each miRNA tested correlate well between the tissue assay and the extracted small RNA fraction. For miR-167a the relative amount is 4.5, which is slightly lower than the tissue assay. Since the fixed tissue sections are taken at similar cross sections, the bulk small RNA tested reflects regions of tissue where miR-167a would be less. We observe expression homogeneity throughout the leaf for miR-159a and miR-396b, while miR-167a target amounts are lower in sections taken toward the edge of the leaf (Fig. S8). The bulk small RNA extracted sample averages over all regions of the leaf and due to the heterogeneous expression levels of miR-167a, this leads to a smaller relative amount when compared to spatially averaged data from the tissue slices in Fig. 4. Therefore, spatial profiling of plant leaves is crucial to capture differing expression patterns reflected across the leaf. Furthermore, we also determined the expected miR-167a amount detected for a gram of leaf tissue using the tissue section and the small RNA enriched fraction (Supplementary Note 1). The expected miR-167a amount for the tissue section is 192.3 amol/mg whereas for bulk the expected miR-167a amount is 229.6 amol/mg. Since the expected amounts agree within 84%, we have shown comparably robust cell lysing methods regardless of the extraction process using the well array. We also perform RT-PCR using small RNA enriched samples (Fig. S9). From Fig. S9, we observe potential cycle amplification bias, which may provide an incorrect PCR readout and may not be accurate when comparing to the tissue assay performance. Therefore, from Fig. 5, we show that we can use multiple sample types (fixed tissue and small RNA enriched) to directly compare the assay performance in the well array.

## Conclusions and outlook

We have developed a spatially resolved and multiplexed technique for the direct capture of miRNA from fixed plant tissue comparable to our platform developed for mammalian tissue. The technique was developed using minimal pre and post processing steps to digest tissue, lyse cells, and capture hybridized miRNA on the surface of a hydrogel-based microwell array. We have shown how our templated-ligation based platform can overcome challenges in plant miRNA methylation and can be optimized to detect unique expression patterns in plant samples, specifically in Col-0 *Arabidopsis thaliana* rosette leaves. Useful spatial observations of different plant miRNA can be detected using our method which have consistent findings to miRNA detected by techniques such as ISH and GUS, but with the added advantages of quantitative and multiplexed target profiling. Furthermore, our optimized tissue assay using the array is validated with commercial small RNA extraction processes, demonstrating the fidelity of our cell lysis technique. In the future, our robust platform for quantitative detection of plant miRNA can be extrapolated to applications such as examining the spatial expression profiles of multiple plant species or studying the effects of different metabolic conditions. Our method can ultimately be used to learn and understand how the developmental and regulatory roles of plants spatially reflects in precise miRNA expression patterns.

## Materials and methods

### Arabidopsis growth and collection

Col-0 *Arabidopsis thaliana* was grown in the greenhouse (Whitehead Institute, MIT) with 16 hours of daily light exposure (120 µmol/m^2^/s) using both fluorescent bulbs and natural light. The temperature was maintained at 22 °C and 50% humidity for 21 days. Potted *Arabidopsis thaliana* was transported indoors and grown under a white light lamp (GooingTop) with 16 h of daily light exposure (120 µmol/m^2^/s) and 23 °C with 75% humidity. *Arabidopsis thaliana* leaves were collected from plants 28 days after germination. All plant leaves were cut 1 cm from the leaf base and handled using forceps. Leaves were weighed before transferring into embedding cassettes (abaxial side facing towards the top of the histology cassette) and immediately stored into fresh 70% ethanol until processing.

### Plant tissue sectioning preparation and processing

Plant leaves are stored in 70% ethanol for one week and transferred to the histology core (Koch Institute, MIT) to prepare for histologic analysis. The plant leaves were 70% ethanol fixed and embedded in paraffin using rapid biopsy processing on the vacuum infiltrating tissue processor (VIP) according to standard histology techniques. After 70% ethanol fixation for one-week processing to paraffin involves dehydration by increasing percentages of ethanol and xylene. The tissue leaves are infiltrated with paraffin and then the leaves are flattened using a tissue tamper (MasterPress) with the abaxial side facing away from the histology cassette. After flattening the section, the tissue is embedded into paraffin blocks. 10 µm sections were taken using a microtome starting from the abaxial side and placed onto microscope slides (Superfrost Plus, Fisherbrand). 10 µm sections were selected because of calculations were performed for expected miRNA amounts per well (Supplementary Note 2). Sections were kept from the center of the tissue because there is greater surface area towards the leaf center. The sections were imaged under a microscope (Aperio AT2, Leica).

### Small RNA enriched extraction

Small RNA was extracted from 28-day old intact plant leaves using the RNAqueous-Micro Total RNA Isolation Kit (Cat. number: AM1931, lot number: 2586176 & 2586171, invitrogen) along with Plant RNA Isolation Aid (Cat. number: AM9690, lot number: 01216055, invitrogen) following the manufacturer’s instructions. Plant leaves were cut 1 cm from the leaf base. Leaves were weighed until ~10 mg of tissue has been added. The leaves were immediately processed using 5 v/v% plant RNA isolation aid and 95 v/v% lysis solution using a manual tissue disruptor (1.5 mL & 2.0 mL pestle, Axygen) until the homogenate appears clear. The lysis process was repeated until 50 mg of tissue has been processed. The lysate was centrifuged for 5 min at 15000 x g and the supernatant was transferred and the volume was recorded. 0.5 volume of 100% ethanol (Pharmco) was mixed with the lysate and passed through a Micro Filter Cartridge Assembly by centrifuging for 30 s at 15000 x g. The cartridge was discarded and the ethanol levels in the flow through was brought up to 1.25 volumes before centrifuging through a second Micro Filter Cartridge Assembly. After centrifugation, the cartridge was rinsed with the two wash solutions provided followed by centrifugation at full speed for 1 min to dry the filter. 60 µL preheated 1xTE buffer was added to the cartridge to collect the small RNA. The collected small RNA was incubated with DNase I for 20 min at 37 °C before adding the DNase inactivating buffer for 2 min at room temperature. The solution was centrifuged at full speed for 1.5 min and the small RNA was collected and stored at -70 °C. Spectrophotometry techniques (NanoDrop One/OneC) were used to determine the concentration and purity (A260/A280 and A260/A230) of the small RNA enriched sample. Samples were diluted to ~1 ng/ul for Small RNA electrophoresis (2100 Bioanalyzer, Agilent) to quantify the miRNA present (Genome Technology Core, Whitehead Institute, MIT).

### Well array device fabrication

Glass slides (BELLCO, Electron Microscopy Sciences) were treated to make the surface hydrophobic with acrylate groups for later creating chemical bonds with the hydrogel posts. Slides were submerged in 1 M NaOH for two hours at room temperature. The slides were rinsed with DI water before acrylating the glass slide surface^31^. Slides were coated in a solution of five parts DI water, three parts acetic acid, and two parts 3-(trimethoxysilyl)propyl methacrylate for two hours at room temperature. The slides were cleaned with methanol and DI water and baked in a vacuum oven at 80 °C for 10 min before storing in a vacuum desiccator. Polydimethylsiloxane (PDMS) (Sylgard 184, Dow) molds were fabricated as described previously^30^. A SU-8 master mold (MicroChem) was prepared using standard photolithography techniques. The SU-8 master mold contains square well features with dimensions of 300 µm × 300 µm and with a depth of 39 µm and 50 µm spacing in between wells. The microfluidic device was casted from the SU-8 wafer mold using PDMS by mixing ten parts elastomer base and one-part curing agent for 5 minutes and left to degas for at least 72 h at room temperature. PDMS molds were removed and 1.5 mm inlets were punched through using a biopsy punch (Miltex, Integra Life Sciences). The PDMS molds were centered on the silanized glass and degassed overnight. The slides were removed from the desiccator and Norland Optical Adhesive 81 (NOA81, Thorlabs) was added to the inlet and loaded through the mold via degas-driven flow for 25 minutes. Slides with molds were UV-cured for six minutes. Molds were removed from the well array slides after curing and the slides were stored under vacuum.

### Hydrogel post fabrication

Functionalized glass slides fixed with cured NOA81 well arrays were submerged in DI water and degassed until air bubbles in the wells were removed. 500 µm spacers were made by stacking three layers of electrical tap (3 M) to a wider glass slide (75 mm × 50 mm, Corning). The NOA81 well array slides were placed with the back of the array facing up and 500 µL of 1×TET buffer (1xTris EDTA and 0.05% Tween 20) was flowed between the array and the spacer where excess buffer was kept in between the array and the spacer for at least 1 minute. PEG-based posts were made from a pre-polymer solution to a final composition of 18% (v/v) PEG diacrylate 700 g/mol, 36% (v/v) PEG 200 g/mol, 4.5% (v/v) 2 hydroxy-2-methylpropiophenone (Darocur 1173 photoinitiator), 41.5% 3xTris EDTA (TE) buffer (pH 8.0). The pre-polymer was cross-linked with acrydite-modified ssDNA probes (Integrated DNA Technologies) for target miRNA capture.

Prior to post fabrication, the wells were viewed using our contact lithography station (Thorlabs)^48^ and initially calibrated with a 2.5 cm × 2.5 cm chrome photomask (Photo Sciences) to evenly position a 1 cm × 1 cm grid composed of 784 40 µm × 40 µm square posts to fit one post per well. Initial calibration to pre-align the posts to the array is performed to minimize subsequent alignment adjustments. To polymerize posts in the well array, 8 µL of pre-polymer solution was spread over the array for 45 seconds and excess solution was aspirated. This step was continued until three washes was completed. 16 µL of pre-polymer solution was loaded onto the array where a PDMS slab (1.5 cm × 1.5 cm) and ~10 g glass weight was placed over the array to seal the device. The array was brought into contact with the chrome photomask and the posts were polymerized through the aspheric condenser lens (Thorlabs, ACL25416U) attached to the UV LED source (Thorlabs, M365L3) to collimate UV light (1.5 mW/cm^2^) through the chrome mask and under the array.

After post polymerization, the glass weight and PDMS were removed where the previous precursor in the array is replaced with a pre-polymer solution containing a different functionalized ssDNA probe. This was repeated using the same washing and polymerization steps until each additional post was added. After post fabrication is complete, four washes of 1xTET (total volume of 1 mL of 1xTET) was flowed between the array and the spacer to rinse excess solution out of the array. Posts were then oxidized by continuously flowing a solution of 500 µM potassium permanganate (Sigma Aldrich) in 0.1 M Tris-HCl buffer (pH 8.8) across the array for 5 minutes. The device was washed four times with 1xTET (total volume of 1 mL) and stored at 4 °C in 1xTET within spacers.

### Well array assay

#### Spatial microRNA assay

The assay involves miRNA hybridization directly from tissue section, ligation of a universal biotinylated linker, labeling with SA-PE, and fluorescence imaging. The device was removed from 4 °C to room temperature and 1×TET was replaced by 350 mM NaCl 1×TET for at least 5 minutes. During this the hybridization buffer is created which consists of 350 mM NaCl 1×TET, 2 w/v% SDS (Sigma Aldrich), 800 µg/mL Proteinase K (New England Biolabs). The glass slide with the tissue section is heated to 45 °C for 2 min while excess paraffin is manually removed around the section.

In total 350 mM NaCl 1×TET was aspirated from the array and 10 µL of the hybridization buffer is loaded to the array and spread around to prime the solution in the wells. Excess hybridization buffer is added to the array and sealed with a tissue section on a glass slide. Clamps (plastic, McMaster Carr) were used to hold the glass slides in place to take brightfield microscopy image. Magnets (Grainger) then sealed the top and bottom of the tissue and array and coverslip sealant (CytoBond) was added to the edge of the glass slide attached to the tissue section to prevent wells drying.

During hybridization the sealed array was heated to 55 °C for 15 min to digest tissues and lyse cells, then heated to 80 °C for 15 min to deactivate enzymes, and held at 55 °C for 3 h for free miRNA to hybridize. After hybridization, the sealant and magnets were removed and the array-tissue section was heated to 55 °C for three minutes before the tissue section was separated and the array was rinsed with preheated 50 mM NaCl 1×TET (R50). The arrays were placed over spacers and washed four times with R50 (total volume of 1 mL).

Ligation is used to covalently join the 3′ miRNA end to the 5′ end of a universal biotinylated linker (Table S1). Ligation solution consisting of 86.9 v/v% 1×TET, 10 v/v% 10×NEBuffer 2 (New England Biolabs), 2.5 v/v% 10 mM ATP (New England Biolabs), 0.4 v/v% 1 mM biotinylated linker (Integrated DNA Technologies), 0.2 v/v% 800 µg/mL T4 DNA ligase (New England Biolabs) is loaded over the array for one hour at room temperature away from light. Excess linker was washed away and the array was rinsed four times with R50 (total volume of 1 mL). The ligated biotinylated linkers are labelled using SA-PE solution which consists of 1 v/v% 1 mg/mL SA-PE (premium grade, invitrogen) and 99 v/v% R50 away from light for one hour at room temperature. Excess SA-PE solution was removed and the array was washed four times with R50 (total volume of 1 mL) and where the array was held in R50 for 30 min for excess SA-PE to diffuse out of posts.

After removing excess R50 from the spacers, an 18 mm × 18 mm microscope cover glass was placed over the array. The cover glass was sealed with clear nail polish (Wet n Wild) and mounted to a glass microscope slide before imaging the array with the Genepix 4400A slide scanner (Molecular Devices). The slides were imaged using a 532 laser set at 100% power, 500 PMT gain, and 100 µm focus position, along with an Alexa568 filter. The scanner resolution was set to a maximum of 5 μm/pixel to prevent any weak auto-fluorescence from the NOA81 wells to interfere with the post signal.

#### Synthetic and bulk extracted small RNA assay

The assay involves miRNA hybridization directly from either synthetic miRNA or plant extracted small RNA enriched samples, ligation of a universal biotinylated linker, and labeling with SA-PE. The assay protocol is identical to the spatial miRNA assay with slight procedural modifications. The hybridization buffer created consists of either synthetic miRNA (Table S1) or plant extracted small RNA enriched samples and made to a final concentration of 350 mM NaCl 1×TET. After loading the hybridization buffer to the array, a clean glass slide was placed over the array to seal the buffer before adding magnets and Cytobond sealant around the clean glass slide. During hybridization, the heating step was held at 55 °C for 2 h. Following hybridization, sealant and magnets were removed and the array was rinsed four times with R50 (total volume of 1 mL) where the ligation, labelling, and imaging steps were identical to the spatial miRNA assay.

### Analysis

Fluorescent images from the slide scanner were processed into TIFF files. Before quantifying the fluorescent signal from each post, we first defined each post to be made up of 8 × 8 pixels. Two wells from the same row were chosen and the pixel position at the upper left corner of each post was recorded. The code applies a convolution to each post and finds the highest averaged intensity generated from 8 × 8 pixels larger than the post size. After analyzing the results, the unprocessed image was evaluated to determine the wells with bright dust particles stuck to posts or fallen posts. The code gave an upper and lower threshold and set these areas to the background signal. For posts that have fallen off, we averaged the signal of the 8 surrounding posts in wells corresponding to the same miRNA target and set the new post signal to the average with ~98% post retention. For data resulting from spatial miRNA assays, we developed a digital mask which defines a region of interest that references the tissue location within the array. The code provides a threshold for tissues with partly filled wells and sets the value outside of the threshold to zero and inside the threshold to one. The digital mask is overlaid over the fluorescent scan after image processing.

### Statistical analysis

All the details of the statistical analysis are further explained in figure descriptions and in their corresponding sections. The reported fluorescence is the signal directly from the scanner subtracted by the negative control signal. The reported amounts use the negative-control subtracted signal and calibration curves (Fig. S5) to get the expected amount. Both the reported fluorescence and miRNA amounts are presented as the mean ± standard deviation. The statistical analysis for leaf sections was conducted using two tailed, unpaired t-test with significance defined by p-values < 0.05. In each analysis, the sample sizes for each test was the number of intact wells within each tissue region.

### RT-PCR and results analysis

In total 161.2 ng of enriched Small RNA was used as an input for reverse transcription (RT) using TaqMan miRNA reverse transcription kit (Applied Biosystems) according to manufacturer instructions. The following TaqMan miRNA probes are used: ath-miR159a (assay ID: 000338, Applied Biosystems), ath-miR396b (assay ID: 000348, Applied Biosystems), ath-miR167a (assay ID: 000367, Applied Biosystems), and snoR85 (assay ID: 001711, Applied Biosystems). snoR85 was selected as an endogenous control (internal control gene)^49^. A negative control omitting small RNA was also reverse transcribed. Tubes with 15 µL RT mixture was placed in a thermal cycler (MultiTherm, Benchmark Scientific) and small RNA is reverse transcribed into cDNA using standard cycling conditions.

cDNA template and negative control produced from reverse transcription along with nuclease free water (NTC) was added to reaction plates (Optical 96-well, MicroAmp) together with universal Master Mix II, no UNG (Applied Biosystems). Three technical replicates were used for each sample tested. PCR was run using compatible equipment (7300 RT-PCR System, Applied Biosystems) using the thermal cycling conditions according to manufacturer instructions and *C*_*T*_ values were analyzed using the 7300 system software. *C*_*T*_ values from RT-PCR were normalized to snoR85 using the ${2}^{-\varDelta \varDelta {C}_{T}}$ method^50^. Relative expression changes were further normalized to the miR-159a fold change values.

## Supplementary information

Supplemental Materials

## Data Availability

All data required to evaluate the conclusions in the paper are in the paper and the Supplementary Materials. Additional data are available from the corresponding author upon reasonable request.

## References

[1] Nobori, T., Oliva, M., Lister, R. & Ecker, J. R. Multiplexed single-cell 3D spatial gene expression analysis in plant tissue using PHYTOMap. *Nat. Plants* **9**, 1026–1033 (2023). 37308583 10.1038/s41477-023-01439-4 PMC10356616

[2] Ma, X., Denyer, T., Javelle, M., Feller, A. & Timmermans, M. C. Genome-wide analysis of plant miRNA action clarifies levels of regulatory dynamics across developmental contexts. *Genome Res.* **31**, 811–822 (2021). 33863807 10.1101/gr.270918.120 PMC8092011

[3] Long, T. A., Brady, S. M. & Benfey, P. N. Systems Approaches to Identifying Gene Regulatory Networks in Plants. *Annu. Rev. cell developmental Biol.* **24**, 81–103 (2008). 10.1146/annurev.cellbio.24.110707.175408 PMC2739012 18616425

[4] Xia, K. et al. The single-cell stereo-seq reveals region-specific cell subtypes and transcriptome profiling in Arabidopsis leaves. *Developmental Cell* **57**, 1299–1310 (2022). 35512702 10.1016/j.devcel.2022.04.011

[5] Saarenpää, S. et al. Spatially resolved host-bacteria-fungi interactomes via spatial metatranscriptomics. *Nat. Biotechnol.* **42**, 1384–1393 (2022). 10.1038/s41587-023-01979-2 PMC11392817 37985875

[6] Pagano, L., Rossi, R., Paesano, L., Marmiroli, N. & Marmiroli, M. miRNA regulation and stress adaptation in plants. *Environ. Exp. Bot.* **184**, 104369 (2021).

[7] Zhang, H., Zhao, Y. & Zhu, J.-K. Thriving under Stress: How Plants Balance Growth and the Stress Response. *Developmental Cell* **55**, 529–543 (2020). 33290694 10.1016/j.devcel.2020.10.012

[8] Zhao, B. et al. Effects of 2-O-methyl nucleotide on ligation capability of T4 DNA ligase. *Acta Biochimica et. Biophysica Sin.* **46**, 727–737 (2014). 10.1093/abbs/gmu058 25022752

[9] Zhao, Y., Mo, B. & Chen, X. Mechanisms that impact microRNA stability in plants. *RNA Biol.* **9**, 1218–1223 (2012). 22995833 10.4161/rna.22034 PMC3583851

[10] Kidner, C. & Timmermans, M. In situ hybridization as a tool to study the role of microRNAs in plant development. *Methods Mol. Biol.* **342**, 159–179 (2006). 16957374 10.1385/1-59745-123-1:159

[11] Mahale, B. M., Fakrudin, B., Ghosh, S. & Krishnaraj, P. U. LNA mediated in situ hybridization of miR171 and miR397a in leaf and ambient root tissues revealed expressional homogeneity in response to shoot heat shock in Arabidopsis thaliana. *J. Plant Biochem. Biotechnol.* **23**, 93–103 (2014).

[12] Munafó, D. B. & Robb, G. B. Optimization of enzymatic reaction conditions for generating representative pools of cDNA from small RNA. *RNA* **16**, 2537–2552 (2010). 20921270 10.1261/rna.2242610 PMC2995414

[13] Chen, F., Fan, C. & Zhao, Y. Inhibitory Impact of 3-Terminal 2-O-Methylated Small Silencing RNA on Target-Primed Polymerization and Unbiased Amplified Quantification of the RNA in Arabidopsis thaliana. *Anal. Chem.* **87**, 8758–8764 (2015). 26244621 10.1021/acs.analchem.5b01683

[14] Yu, Y., Jia, T. & Chen, X. The ‘how’ and ‘where’ of plant microRNAs. *New Phytol.* **216**, 1002–1017 (2017). 10.1111/nph.14834 PMC6040672 29048752

[15] Giacomello, S. & Lundeberg, J. Preparation of plant tissue to enable Spatial Transcriptomics profiling using barcoded microarrays. *Nat. Protoc.* **13**, 2425–2446 (2018). 30353173 10.1038/s41596-018-0046-1

[16] Ghawana, S. et al. An RNA isolation system for plant tissues rich in secondary metabolites. *BMC Res. Notes* **4**, 85 (2011). 21443767 10.1186/1756-0500-4-85 PMC3079660

[17] Sarkar, P., Bosneaga, E. & Auer, M. Plant cell walls throughout evolution: Towards a molecular understanding of their design principles. *J. Exp. Bot.* **60**, 3615–3635 (2009). 19687127 10.1093/jxb/erp245

[18] Martinho, C. et al. Dissection of miRNA Pathways Using Arabidopsis Mesophyll Protoplasts. *Mol. Plant* **8**, 261–275 (2015). 25680775 10.1016/j.molp.2014.10.003

[19] Borges, F. et al. FACS-based purification of Arabidopsis microspores, sperm cells and vegetative nuclei. *Plant Methods* **8**, 44 (2012). 23075219 10.1186/1746-4811-8-44 PMC3502443

[20] Grolmusz, V. K. et al. Fluorescence activated cell sorting followed by small RNA sequencing reveals stable microRNA expression during cell cycle progression. *BMC genomics* **17**, 412 (2016). 27234232 10.1186/s12864-016-2747-6 PMC4884355

[21] Chen, K., Chen, J., Pi, X., Huang, L.-J. & Li, N. Isolation, Purification, and Application of Protoplasts and Transient Expression Systems in Plants. *Int. J. Mol. Sci.* **24**, 16892 (2023). 38069215 10.3390/ijms242316892 PMC10706244

[22] Wang, J. et al. An Efficient and Universal Protoplast Isolation Protocol Suitable for Transient Gene Expression Analysis and Single-Cell RNA Sequencing. *Int. J. Mol. Sci.* **23**, 3419 (2022). 35408780 10.3390/ijms23073419 PMC8998730

[23] Kilic, T., Erdem, A., Ozsoz, M. & Carrara, S. microRNA biosensors: Opportunities and challenges among conventional and commercially available techniques. *Biosens. Bioelectron.* **99**, 525–546 (2018). 28823978 10.1016/j.bios.2017.08.007

[24] Koshiol, J., Wang, E., Zhao, Y., Marincola, F. & Landi, M. T. Strengths and limitations of laboratory procedures for microRNA detection. *Cancer Epidemiol. Biomark**. Prev*. **19**, 907–911 (2010). 10.1158/1055-9965.EPI-10-0071 PMC2852469 20332265

[25] Wójcik, A. M., Mosiolek, M., Karcz, J., Nodine, M. D. & Gaj, M. D. Whole Mount in situ Localization of miRNAs and mRNAs During Somatic Embryogenesis in Arabidopsis. *Front. Plant Sci.* **9**, 1277–1290 (2018). 10.3389/fpls.2018.01277 PMC6131960 30233621

[26] Chen, A. et al. Spatiotemporal transcriptomic atlas of mouse organogenesis using DNA nanoball-patterned arrays. *Cell* **185**, 1777–1792 (2022). 35512705 10.1016/j.cell.2022.04.003

[27] Yin, R., Xia, K., & Xu, X. Spatial transcriptomics drives a new era in plant research. *The Plant J.* **116**, 1571–1581 (2023). 10.1111/tpj.16437 37651723

[28] Deng, Y., Bai, Z. & Fan, R. Microtechnologies for single-cell and spatial multi-omics. *Nat. Rev. Bioeng.* **1**, 769–784 (2023).

[29] McKellar, D. W. et al. Spatial mapping of the total transcriptome by in situ polyadenylation. *Nat. Biotechnol.* **41**, 513–520 (2023). 36329320 10.1038/s41587-022-01517-6 PMC10110464

[30] Nagarajan, M. B., Tentori, A. M., Zhang, W. C., Slack, F. J. & Doyle, P. S. Spatially resolved and multiplexed MicroRNA quantification from tissue using nanoliter well arrays. *Microsyst. Nanoengineering* **6**, 51 (2020). 10.1038/s41378-020-0169-8 PMC7211184 32419951

[31] Tentori, A. M. et al. Quantitative and multiplex microRNA assays from unprocessed cells in isolated nanoliter well arrays. *Lab a Chip* **18**, 2410–2424 (2018). 10.1039/c8lc00498f PMC6081239 29998262

[32] Chapin, S. C., Appleyard, D. C., Pregibon, D. C., & Doyle, P. S. Rapid microRNA Profiling on Encoded Gel Microparticles. *Angew. Chem. Int. Ed.* **50**, 2289–2293 (2011). 10.1002/anie.201006523 PMC4104285 21351338

[33] Lee, H., Shapiro, S. J., Chapin, S. C. & Doyle, P. S. Encoded Hydrogel Microparticles for Sensitive and Multiplex microRNA Detection Directly from Raw Cell Lysates. *Anal. Chem.* **88**, 3075–3081 (2016). 26863201 10.1021/acs.analchem.5b03902

[34] Behlke, M. A. Chemical Modification of siRNAs for In Vivo Use. *Oligonucleotides* **18**, 305–320 (2008). 19025401 10.1089/oli.2008.0164

[35] Yu, B. et al. Methylation as a Crucial Step in Plant microRNA Biogenesis. *Science* **307**, 932–935 (2005). 15705854 10.1126/science.1107130 PMC5137370

[36] Bullard, D. R. & Bowater, R. P. Direct comparison of nick-joining activity of the nucleic acid ligases from bacteriophage T4. *Biochem. J.* **398**, 135–144 (2006). 16671895 10.1042/BJ20060313 PMC1525015

[37] Nilsson, M., Antson, D.-O., Barbany, G. & Landegren, U. RNA-templated DNA ligation for transcript analysis. *Nucleic Acids Res.* **29**, 578–581 (2001). 11139629 10.1093/nar/29.2.578 PMC29667

[38] Pinon, V., Ravanel, S., Douce, R. & Alban, C. Biotin Synthesis in Plants. The First Committed Step of the Pathway Is Catalyzed by a Cytosolic 7-Keto-8-Aminopelargonic Acid Synthase. *Plant Physiol.* **139**, 1666–1676 (2005). 16299174 10.1104/pp.105.070144 PMC1310550

[39] Koonjul, P. K., Brandt, W. F., Farrant, J. M. & Lindsey, G. G. Inclusion of polyvinylpyrrolidone in the polymerase chain reaction reverses the inhibitory effects of polyphenolic contamination of RNA. *Nucleic Acids Res.* **27**, 915–916 (1999). 9889293 10.1093/nar/27.3.915 PMC148267

[40] Rashid, R. et al. Novel Use for Polyvinylpyrrolidone as a Macromolecular Crowder for Enhanced Extracellular Matrix Deposition and Cell Proliferation. *Tissue Eng. Part C., Methods* **20**, 994–1002 (2014). 24665935 10.1089/ten.tec.2013.0733 PMC4241873

[41] Eamens, A. L., Smith, N. A., Curtin, S. J., Wang, M.-B. & Waterhouse, P. M. The Arabidopsis thaliana double-stranded RNA binding protein DRB1 directs guide strand selection from microRNA duplexes. *RNA* **15**, 2219–2235 (2009). 19861421 10.1261/rna.1646909 PMC2779670

[42] Hou, N. et al. Epigenetic regulation of miR396 expression by SWR1-C and the effect of miR396 on leaf growth and developmental phase transition in Arabidopsis. *J. Exp. Bot.* **70**, 5217–5229 (2019). 31198943 10.1093/jxb/erz285 PMC6793462

[43] Reinhart, B. J., Weinstein, E. G., Rhoades, M. W., Bartel, B. & Bartel, D. P. MicroRNAs in plants. *Genes Dev.* **16**, 1616–1626 (2002). 12101121 10.1101/gad.1004402 PMC186362

[44] Debernardi, J. M., Rodriguez, R. E., Mecchia, M. A. & Palatnik, J. F. Functional Specialization of the Plant miR396 Regulatory Network through Distinct MicroRNA–Target Interactions. *PLOS Genet.* **8**, e1002419 (2012). 22242012 10.1371/journal.pgen.1002419 PMC3252272

[45] Farmer, E., Mousavi, S. & Lenglet, A. Leaf numbering for experiments on long distance signalling in Arabidopsis. *Protoc. Exch*. 1–7 (2013).

[46] Satterlee, J. W. & Scanlon, M. J. Coordination of Leaf Development Across Developmental Axes. *Plants* **8**, 433 (2019). 31652517 10.3390/plants8100433 PMC6843618

[47] Borsuk, A. M., Roddy, A. B., Théroux-Rancourt, G. & Brodersen, C. R. Structural organization of the spongy mesophyll. *New Phytol.* **234**, 946–960 (2022). 10.1111/nph.17971 PMC9303971 35037256

[48] Le Goff, G. C., Lee, J., Gupta, A., Hill, W. A. & Doyle, P. S. High-Throughput Contact Flow Lithography. *Adv. Sci*. **2**, 1500149 (2015). 10.1002/advs.201500149 PMC5115321 27980910

[49] Marker, C. et al. Experimental RNomics: Identification of 140 Candidates for Small Non-Messenger RNAs in the Plant Arabidopsis thaliana. *Curr. Biol.* **12**, 2002–2013 (2002). 12477388 10.1016/s0960-9822(02)01304-0

[50] Livak, K. J. & Schmittgen, T. D. Analysis of Relative Gene Expression Data Using Real- Time Quantitative PCR and the 2CT Method. *Methods* **25**, 402–408 (2001). 11846609 10.1006/meth.2001.1262

