# Supplementary material for: Quantitative and spatially resolved detection of multiplexed microRNA from plant tissue via hybridization to hydrogel-bound DNA probes in nanoliter well arrays
Source: Microsyst Nanoeng. 2024 Oct 8;10:142. doi: 10.1038/s41378-024-00785-3 (PMC11458878; doi:10.1038/s41378-024-00785-3)
Supplement: Supplementary file 1 — Supplemental Materials [file 41378_2024_785_MOESM1_ESM.docx]

**Supplementary Materials**

Quantitative and Spatially Resolved Detection of Multiplexed MicroRNA from Plant Tissue via Hybridization to Hydrogel-bound DNA Probes in Nanoliter Well Arrays

Jennifer Fang and Patrick S. Doyle*

Department of Chemical Engineering, Massachusetts Institute of Technology, Cambridge, MA, 02139, USA

*Corresponding author. Email: [pdoyle@mit.edu](mailto:pdoyle@mit.edu)

**Contents**

Fig. S1. Optimization of tissue assay using serial sections and varying paraffin removal techniques.

Fig. S2. Optimization of tissue assay using serial sections and varying Proteinase K concentration.

Fig. S3. Optimization of tissue assay using serial sections and varying digestion times.

Fig. S4. Signal cross-talk for three endogenous miRNA used in plant tissue assay.

Fig. S5. Calibration curves and corresponding LOD developed for three endogenous miRNA used in plant tissue assay.

Fig. S6. Unprocessed supporting images for Fig. 4. in main text.

Fig. S7. Nanodrop and Agilent 2100 Bioanalyzer results for sRNA extraction.

Fig. S8. Multiplexed miRNA from same Arabidopsis Thaliana leaves using non-adjacent sections.

Fig. S9. Relative endogenous miRNA amounts from small RNA enriched samples in *Arabidopsis thaliana* leaves using RT-PCR.

Table S1. Nucleic acid sequences for probes and targets.

Supplementary Note 1. Comparison of miRNA measured in tissue assay to small RNA extraction using nanoliter well array.

Supplementary Note 2. Sectioning thickness using estimates of miRNA amounts captured per well.


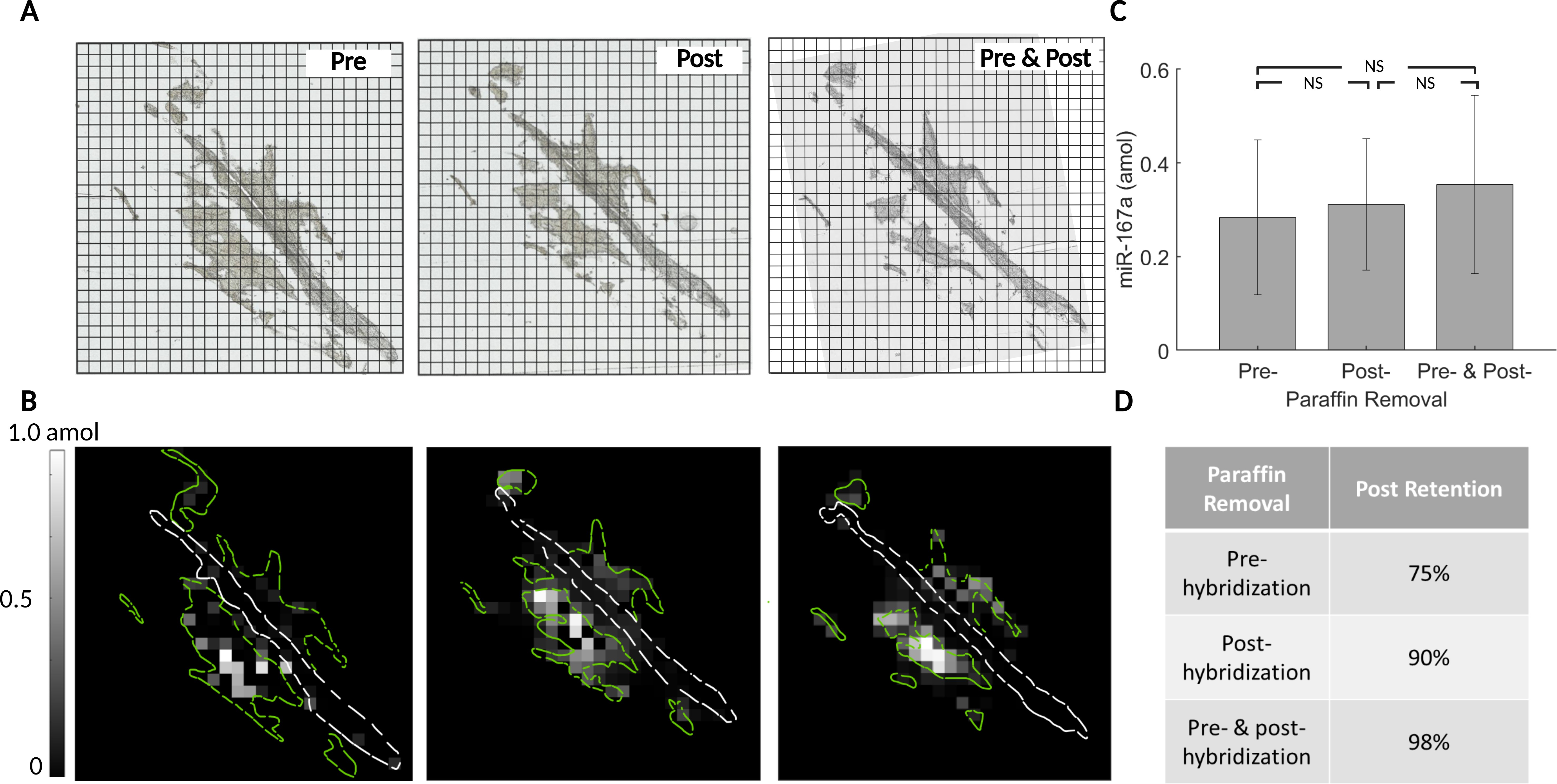


Figure S1: **Optimization of tissue assay using serial sections and varying paraffin removal techniques.** (A) Location of each of the 3 tissue sections within the array where each box represents a 300 µm x 300 µm well, with well spacing separated by 50 µm. The paraffin removal procedure during pre-hybridization heats the tissue to 45*°*C for 2 minutes to gently remove excess paraffin around the tissue. Post-hybridization paraffin removal heats the sandwiched array and tissue to 55 *°*C for 3 minutes to remove excess paraffin in the wells. (B) Heatmaps for miR-167a of the three serial sections after performing the tissue assay. Each pixel in the heatmap corresponds to the amount of miRNA detected in a well. Reported values are negative control (cel-miR-54) subtracted. (C) Quantitative plot for each of the three tissue sections. Values are averaged from the heatmap after applying a mask to threshold pixel values with partly-filled tissue sections. Each value represents the mean, error bars represent one standard deviation. NS indicates not significant using unpaired t-tests. (D) Post retention for the three sections was performed by averaging the number of fallen miR-167a posts within the tissue section over all miR-167a posts within the tissue section.


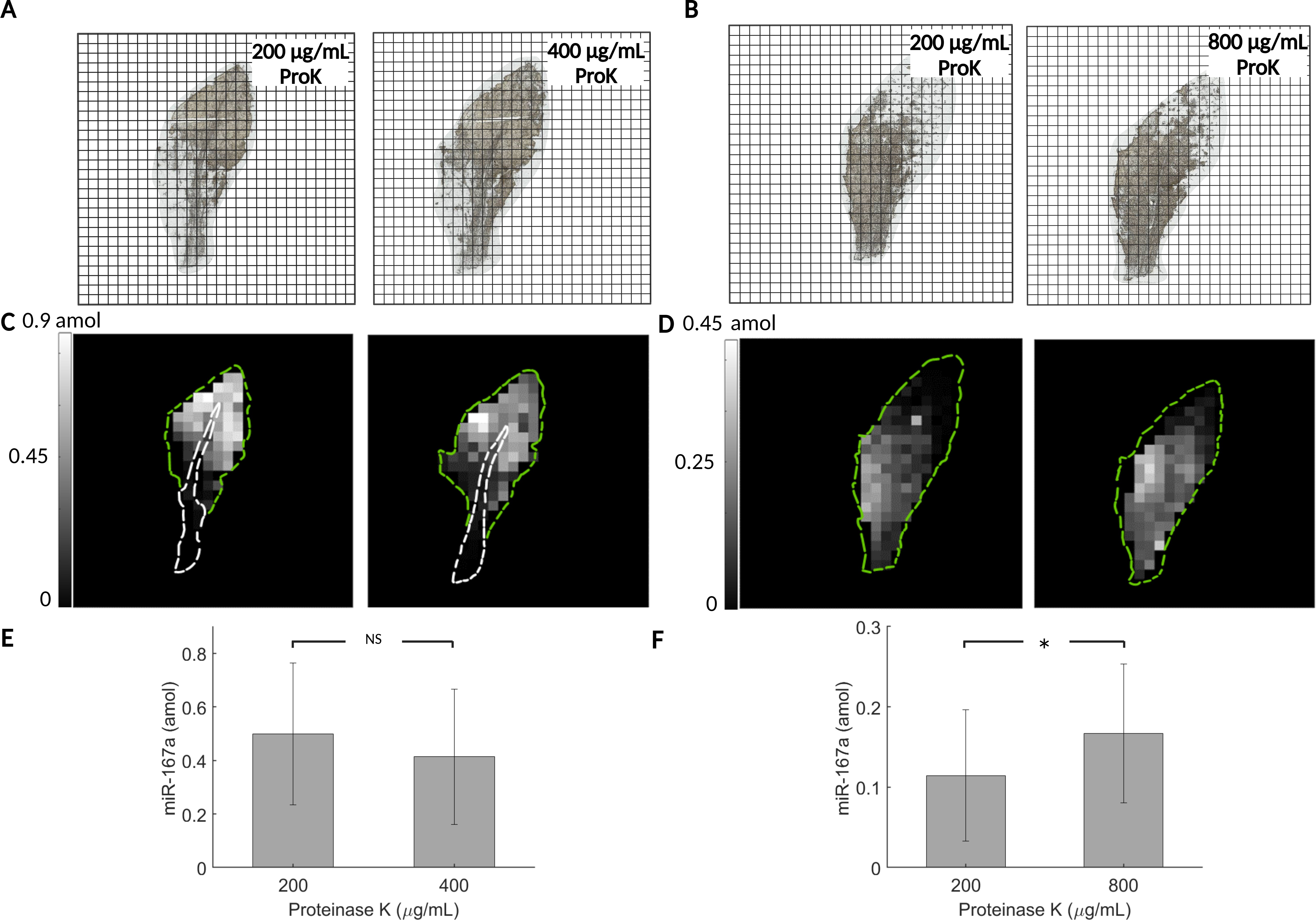


Figure S2: **Optimization of tissue assay using serial sections and varying Proteinase K concentration.** (A/B) Location of each of the 2 adjacent tissue sections within the array where each box represents a 300 µm x 300 µm well, with well spacing separated by 50 µm. 200 µg/mL, 400 µg/mL, or 800 µg/mL Proteinase K is added to the lysis buffer. (C/D) Heatmaps for miR-167a of the 2 adjacent sections after performing the tissue assay. Each pixel in the heatmap corresponds to the amount of miRNA detected in a well. Re- ported values are negative control (cel-miR-54) subtracted. (E/F) Quantitative plot for each of the 2 adjacent tissue sections. Values are averaged from the heatmap after applying a mask to threshold pixel values with partly-filled tissue sections. Each value represents the mean, error bars represent one standard deviation. NS indicates not significant, *(p *<* 0.05) indicates statistical significance using unpaired t-tests.


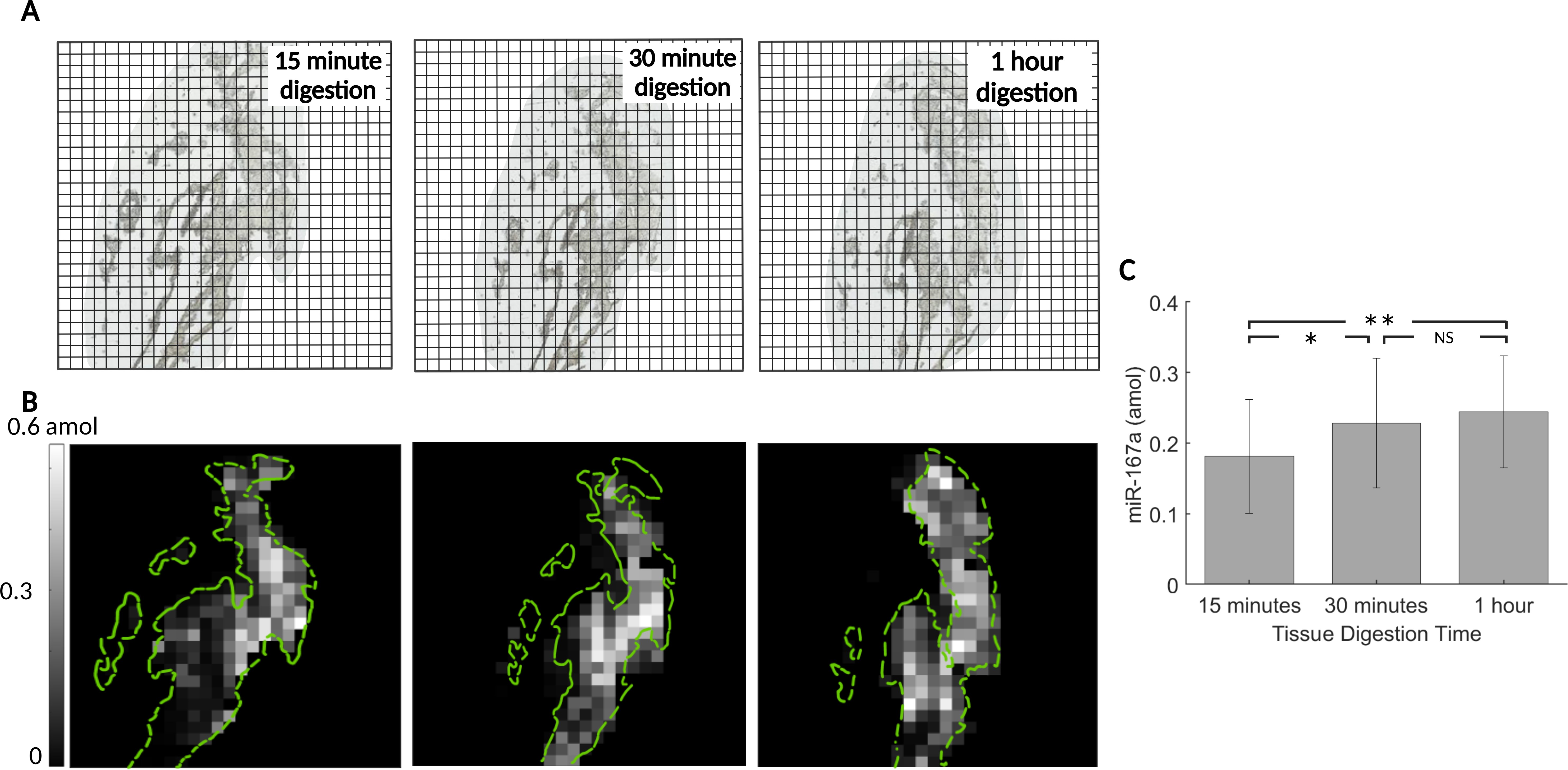


Figure S3: **Optimization of tissue assay using serial sections and varying digestion times.** (A) Location of each of the 3 adjacent tissue sections within the array where each box represents a 300 µm x 300 µm well, with well spacing separated by 50 µm. Tissue sections were subjected to 15 minutes, 30 minutes, or 1 hour during digestion (55*°*C) prior to 85*°*C Proteinase K deactivation and 3-hour hybridization at 55*°*C. (B) Heatmaps for miR-167a of the 3 adjacent sections after performing the tissue assay. Each pixel in the heatmap corresponds to the amount of miRNA detected in a well. Reported values are negative control (cel-miR-54) subtracted. (C) Quantitative plot for each of the 3 adjacent tissue sections. Values are averaged from the heatmap after applying a mask to threshold pixel values with partly-filled tissue sections. Each value represents the mean, error bars represent one standard deviation. NS indicates not significant, *(p *<* 0.05), **(p *<* 0.01) indicates statistical significance using unpaired t-tests.


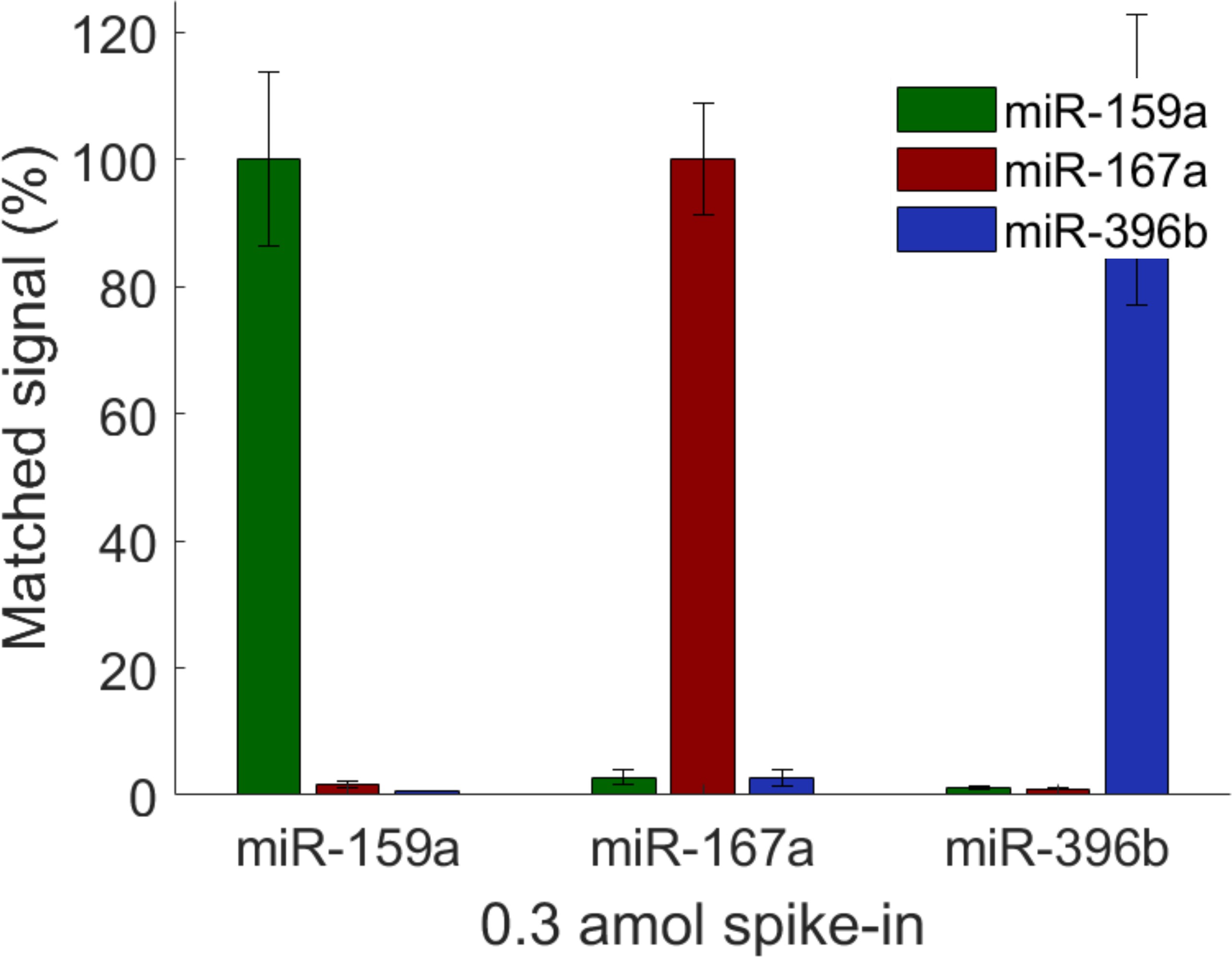


Figure S4: **Signal cross-talk for three endogenous miRNA used in plant tissue assay.** Five posts were polymerized in each device. This includes one negative control (cel-miR-54), one internal control (0.2 µM biotinylated post), and three endogenous plant miRNA targets (sequences provided in Table S1). Neat synthetic samples containing 0.3 amol of one endogenous miRNA target was added and sealed with a cover glass and magnets before miRNA hybridization. Assay was repeated for each unique endogenous miRNA target. Values are control (0 amol) subtracted from each miRNA signal. Matched signal is reported as the control-subtracted signal of each post normalized to the signal of the corresponding target miRNA added.


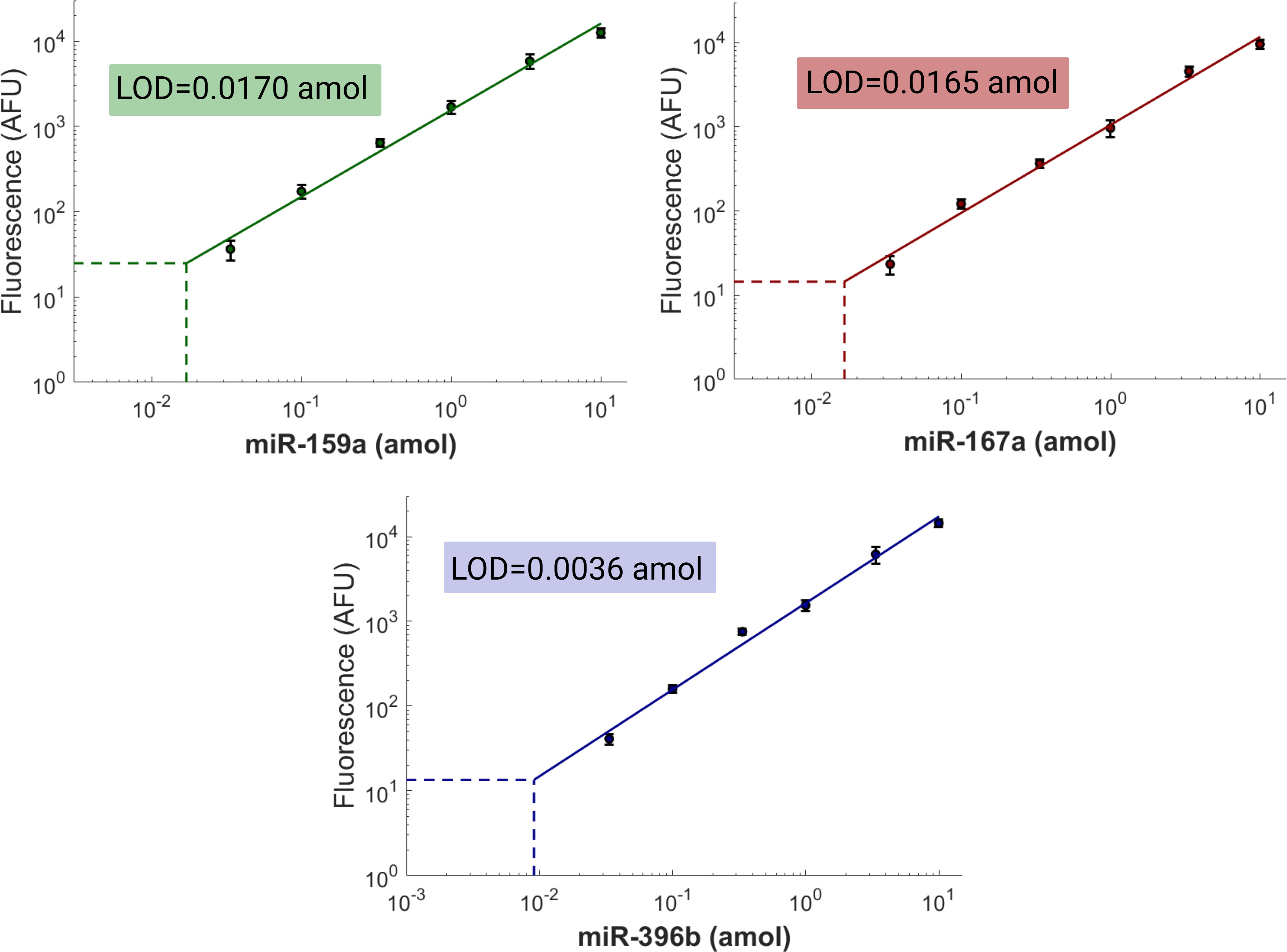


Figure S5: **Calibration curves and corresponding LOD developed for three endogenous miRNA used in plant tissue assay.** Five posts were polymerized in each device. This includes one negative control (cel-miR-54), one internal control (0.2 µM biotinylated post), and three endogenous plant miRNA targets (sequences provided in Table S1). The calibration curves were constructed by adding in neat synthetic miRNA targets and sealing the array with a cover glass and magnets before miRNA hybridization. Reported values are control (0 amol) subtracted from each corresponding miRNA signal. Error bars represent a standard deviation. Dashed lines show the LOD as three times the standard deviation of the control (0 amol) signal.


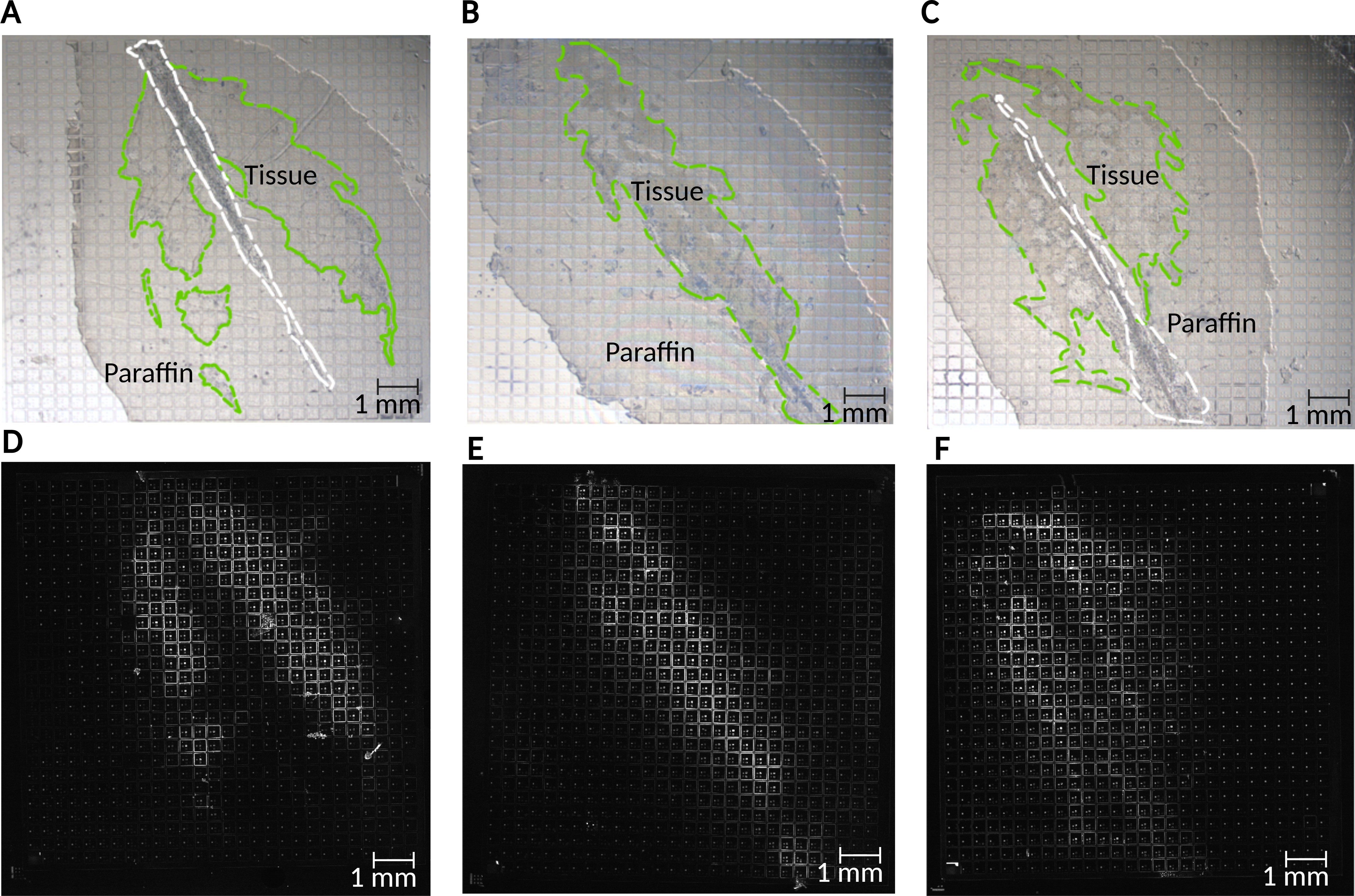


Figure S6: **Unprocessed supporting images for Fig. 4. in main text.** (A) Image taken prior to assay from Fig. 4A-C, with array and tissue section clamped together. (B) Image taken prior to assay (Fig. 1B) from Fig. 4D-F, with array and tissue section clamped together. (C) Image taken prior to assay (Fig. 1B) from Fig. 4G-I, with array and tissue section clamped together. (A-C) Excess paraffin was removed around the tissue section and the tissue sections are outlined. (D) Fluorescent image of the array after the assay from Fig. 4A-C. (E) Fluorescent image of the array after the assay from Fig. 4D-F. (F) Fluorescent image of the array after the assay from Fig. 4G-I.


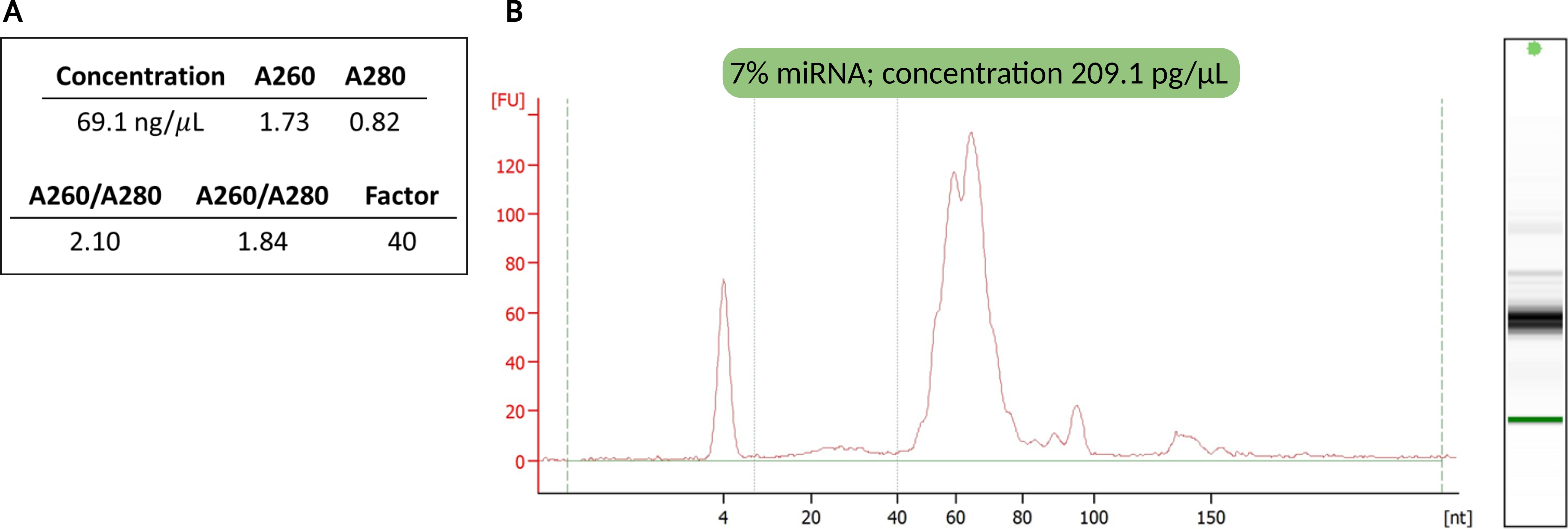


# Figure S7: Nanodrop and Agilent 2100 Bioanalyzer results for sRNA extraction. (A) Nucleic acid concentration and absorbance maxima using Nanodrop are at 260 mm and 280 mm respectively. Nucleic acids (RNA) are considered pure when A260/A280 is *>*2.0. (B) small RNA quantification using the Agilent 2100 Bioanalyzer. Small RNA electrophoresis resolves nucleic acids in the range of 6 to 150 nt. miRNA concentration is found by integrating the peak between 10 to 40 nt where the average miRNA size is 26 nt.


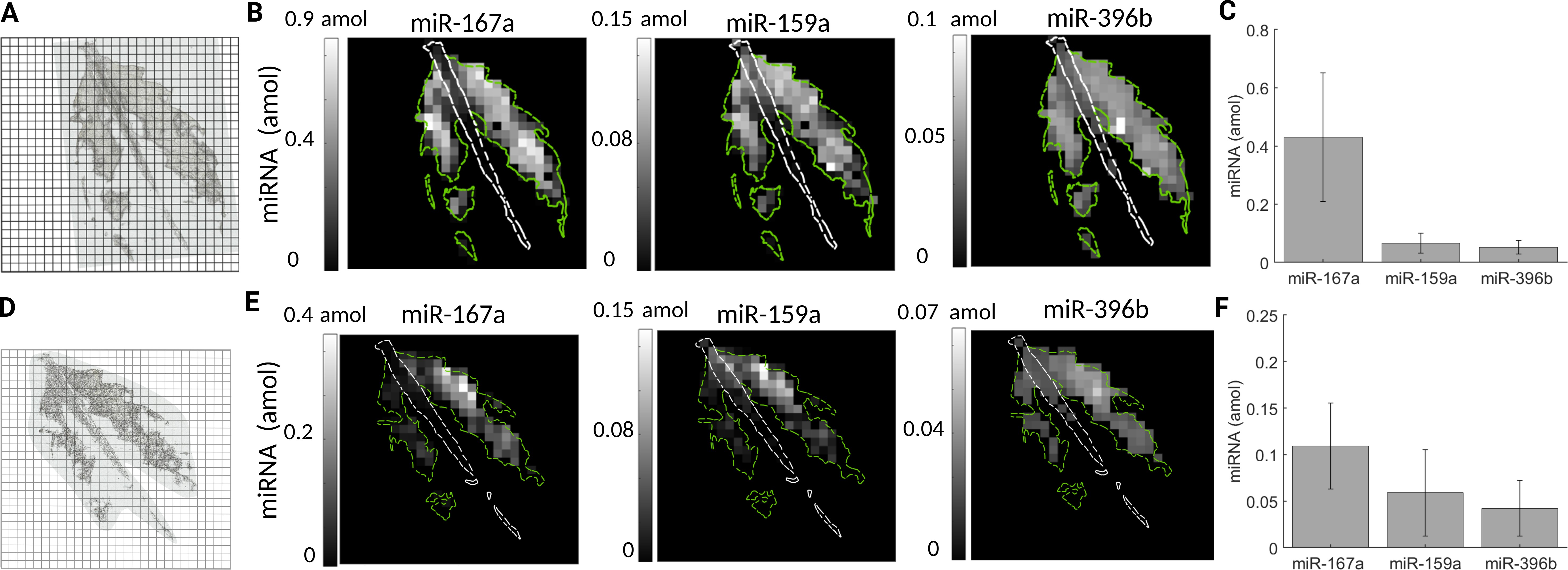


Figure S8: **Multiplexed miRNA from same *Arabidopsis Thaliana* leaf using non- adjacent sections.** (A-C) Multiplex tissue assay using data taken from Fig. 4 A-C where section is taken at the center of the leaf. (D-F) Multiplex tissue assay using section taken from the same *Arabidopsis Thaliana* leaf taken towards the edge of the leaf. (D) Location of tissue section within the array where each box represents a 300 µm x 300 µm well, with well spacing separated by 50 µm. (E) Representative heatmaps for three plant miRNAs detected after performing assay after optimization. Each reported value is negative control (cel-miR-54) subtracted and relates to the miRNA captured in its corresponding 300 µm x 300 µm well. (F) Quantitative plots for each plant miRNA in the multiplexed assay from Fig. S8E. Values are averaged from the heatmap after applying a threshold to pixel values with partly-filled tissue sections. Each value represents the mean and error bars represent one standard deviation.


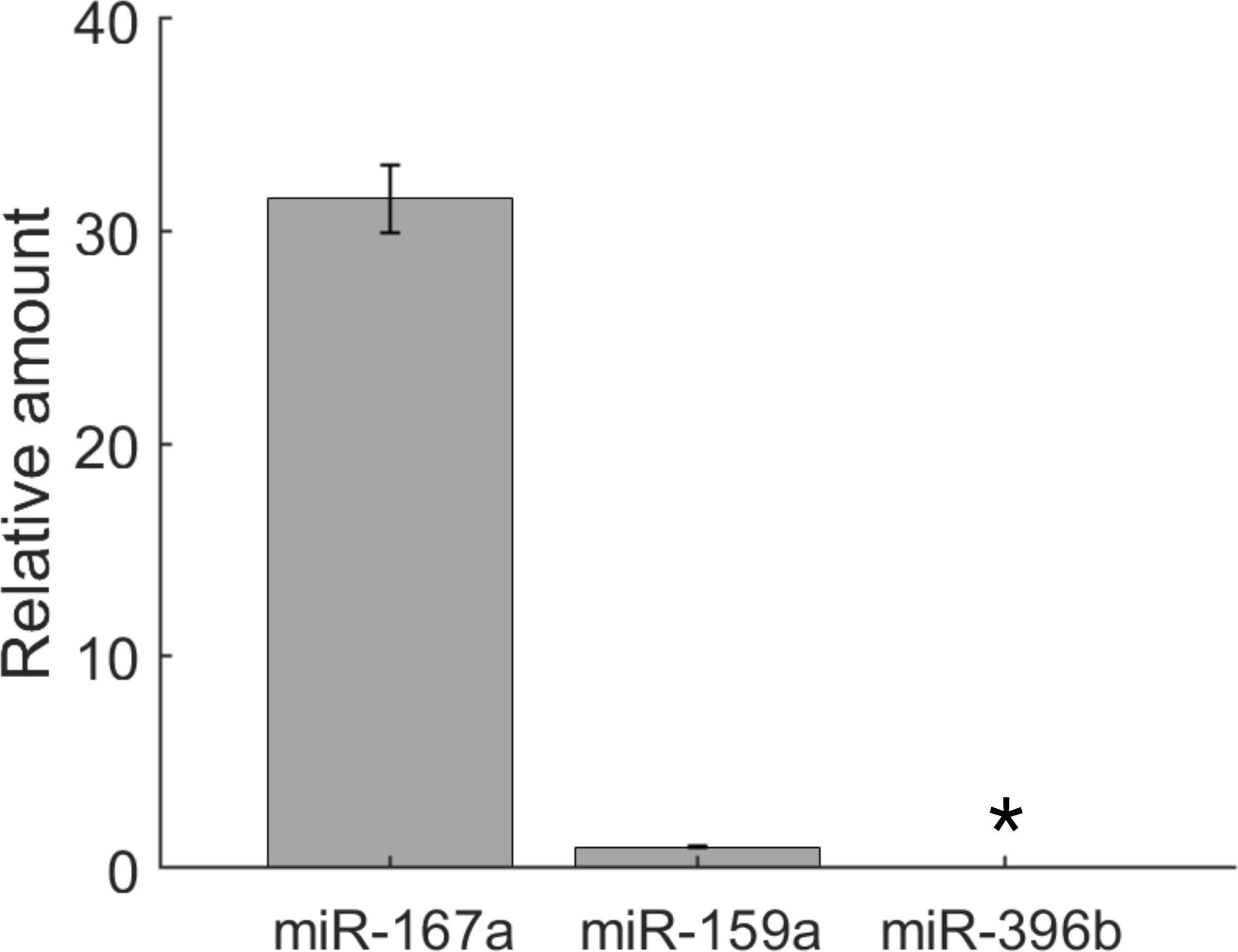


Figure S9: **Relative endogenous miRNA amounts from small RNA enriched samples in *Arabidopsis thaliana* leaves using RT-PCR.** Small RNA enriched samples were used as an input for RT-PCR. SnoR85 served as a housekeeping gene and three technical replicates were used for each miRNA in the experiment. The averaged value was normalized to endogenous control snoR85 to determine the relative gene expression changes through the 2*^−^*^∆∆^*^C^_T_* method.^1^ The normalized fold change values were then normalized to miR-159a. Error bars represent one standard deviation. For miR-396b, the * indicates the relative amount is much less than one (relative amount=0.015). The relative expression patterns between RT-PCR and the well array assay (both small RNA enriched and tissue section) are similar for the endogenous miRNA. However, the relative amounts in RT-PCR are larger than the well array assay.

We also performed RT-PCR using the bulk small RNA fraction to compare to the well array method results from Fig. 5. We used the same small RNA sample collected from 4-week-old Col-0 *Arabidopsis thaliana* leaves which were also used in the bulk assay (Fig. 5B). We used TaqMan miRNA assays for RT-PCR with multiplexing using three endogenous miRNA targets (miR-167a, miR-159a, miR-396b). The resulting cycle threshold values were normalized using the 2*^−^*^∆∆^*^C^_T_* method^1^ with snoR85 (small non-coding RNA) as the endogenous internal control.^2^ The fold change miRNA amounts were then normalized to miR-159a and reported as a relative amount (Fig. S9) since we have shown miR-159a has an intermediate abundance between miR-167a and miR-396b (Fig. 4), which allows both miR-167a and miR-396b to be easily visualized. While the relative expression of all target trends similar to Fig. 5, the overall relative amounts are larger using RT-PCR compared to the tissue section or bulk assay from the well array. Similarly, to prior work, the fold change measured by RT-PCR was up to an order of magnitude greater than the tissue assay performed on the well array.^3^ Deviations in relative amount from the well array method can be attributed to the RNA preparation involved in the enrichment of small RNA. RT-PCR using enriched small RNAs were chosen to eliminate sample differences between the RT-PCR and well array assay so that variability would be inherent to the assay performance. However, small RNA input produces variable results compared to total RNA input which is reflected using target-specific miRNA (12%-35% yield) in RT-PCR which can bias the relative amounts.^4^ Furthermore, RT-PCR can have assay variations through adaptor ligation and cycle amplification biases.^5^ These are directed towards sequence specific primers which can vary the amplification efficiencies. Therefore, evaluating the tissue assay performance may not be accurate when comparing to RT-PCR.

Table S1: **Nucleic acid sequences for probes and targets.** All DNA probes have a 5’ Acrydite modification and a 3’ inverted dT modification. The biotinylated linker has a 5’ phosphate modification and a 3’ biotin modification. miR-159a (methylated) target has a 3’ 2’-O-Methyl modification. Concentrations listed are the final working concentration. RNA sequences were used to generate calibration curves and ligase efficiency data using methyl substitutions.


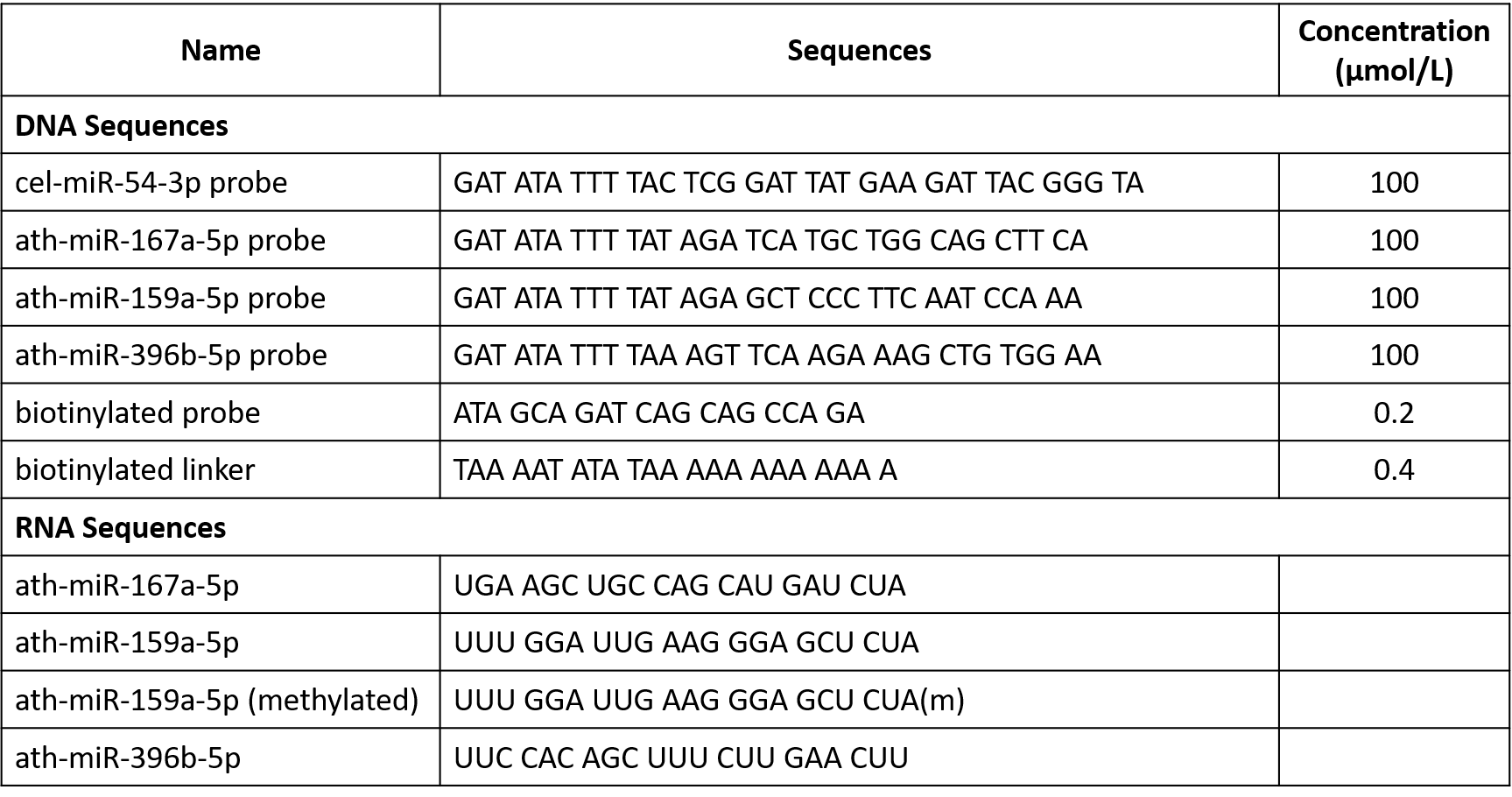


# Supplementary Note 1. Comparison of miRNA measured in tissue assay to small RNA extraction using well array.

We can determine the amount of miRNA captured from the weight of starting plant material from two different techniques using the well array. Using the tissue sections from ethanol fixed, paraffin embedded *Arabidopsis thaliana* plant leaves we find that each leaf weighs 10 mg and approximately 30 sections are taken for each leaf (0.33 mg/leaf). Since the starting amount is distributed across wells, we apply the digital mask to the sections in Fig. 4 and count the number of wells which contains tissue to find approximately 125 wells with tissue. So, for each well we expect 2*.*6 *×* 10^−3^ mg/well. After running the tissue assay and averaging the amount of miR-167a in each well in that contains tissue in Fig. 4, we get 0.5 amol miR-167a/well. We would expect:


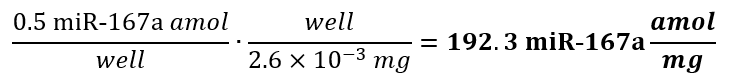


(1)

For the small RNA extracted from fresh leaves using the RNAqueous isolation kit, 50.4 mg of starting leaf tissue was used during cell lysis and small RNA collection. 40 µL was used to elute the small RNA. For each bulk RNA assay we dilute 6.5 µL of small RNA to 50 µL for the hybridization buffer. Furthermore, for each well array only a fraction of the hybridization buffer will fill the wells. We can approximate the total occupied volume as 300 µm × 300 µm × 40 µm × 784 wells=2.8 µL. Therefore, the tissue amount used in bulk is 0.458 mg tissue used (per assay). Since the starting amount is distributed across 784 wells so we would expect 5.85 × 10^−4^ mg/well. After running the bulk assay and averaging the amount of miR-167a, we get 0.1343 amol miR-167a/well. We would expect:


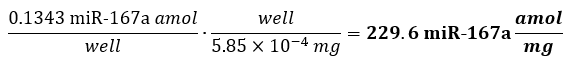


(2)

The expected amount per mg of tissue between ethanol fixed, paraffin embedded leaves and small RNA extracted from leaves agree within 84%, despite the different extraction

methods. This indicates a fairly robust cell lysis process during hybridization. Potential causes for the discrepancy in expected amounts can arise from nucleic acid losses using spin columns. Ineffective nucleic acid binding or washing on the spin column can reduce the amount of sample before elution, thereby reducing the working concentration. There are also biases in sectioning fixed leaves since extracting small RNA from fresh tissue requires using all of the plant material. When sectioning fixed plant leaves, we choose to recover the greatest surface area so the center of the leaves is selected. Since we do not sample the entire leaf during sectioning, we would expect differences in the amount of miRNA captured across the leaf.

# Supplementary Note 2. Sectioning thickness using estimates of miRNA amounts captured per well.

When determining the optimal section thickness of ethanol fixed, paraffin embedded leaves needed for miRNA capture, we need to estimate the expected miRNA copies per well. In previous work using the well arrays to detect miRNA from formalin fixed, paraffin embedded tissue, the well feature sizes of 300 µm × 300 µm × 40 µm were selected along with 5 µm tumor sections to detect between 2 to 5 amol miR-21 per well.^3^ In plants, the miRNA copy numbers per cell are less abundant and the cells are larger than mammalian systems. Therefore, to estimate the expected number of copies per well we first found the average number of cells per well. Since plant cells vary between 10 to 100 µm in size, we can take the intermediate cell value. Since the well feature size is 300 µm × 300 µm, the intermediate number of cells per well is 450. Second, in literature plant miRNA copies per cell for miR- 159a can be approximated as 390 using the fresh plant copy number per gram and knowing the weight of each cell (1 × 10^−9^ g/cell).^6^ Provided that the plant sections are 10 µm thick and the average plant cell height is 50 µm, we expect 0.058 amol miR-159a per well. This value is above the LOD for miR-159a (0.0170 amol/well). Furthermore, when using 10 µm Arabidopsis thaliana plant leaf sections and performing the tissue assay on the well array the measured amount from tissue is 0.07 amol miR-159a per well as shown in Fig. 4, demonstrating comparable agreement between the expected and measure value.

**References**

1. Livak, K. J.; Schmittgen, T. D. Analysis of Relative Gene Expression Data Using Real- Time Quantitative PCR and the 2CT Method. *Methods* **2001**, *25*, 402–408.
2. Marker, C.; et al. Experimental RNomics: Identification of 140 Candidates for Small Non-Messenger RNAs in the Plant Arabidopsis thaliana. *Current Biology* **2002**, *12*, 2002–2013.
3. Nagarajan, M. B.; Tentori, A. M.; Zhang, W. C.; Slack, F. J.; Doyle, P. S. Spatially re- solved and multiplexed MicroRNA quantification from tissue using nanoliter well arrays. *Microsystems & Nanoengineering* **2020**, *6*, 51.
4. Redshaw, N.; et al. A Comparison of miRNA Isolation and RT-qPCR Technologies and their Effects on Quantification Accuracy and Repeatability. *BioTechniques* **2013**, *54*, 155–164.
5. Ruijter, J. M.; et al. Amplification efficiency: linking baseline and bias in the analysis of quantitative PCR data. *Nucleic Acids Research* **2009**, *37*, e45.
6. Xie, W.; Melzig, M. F. The Stability of Medicinal Plant microRNAs in the Herb Prepa- ration Process. *Molecules* **2018**, *23*, 919.
